# Intracellular *Staphylococcus aureus* Infection Decreases Milk Protein Synthesis by Preventing Amino Acid Uptake in Bovine Mammary Epithelial Cells

**DOI:** 10.3389/fvets.2021.756375

**Published:** 2021-11-16

**Authors:** Yuhao Chen, Yuze Ma, Qiang Ji, Xiaoru Yang, Xue Feng, Ruiyuan Yao, Xiaoou Cheng, Tingting Li, Yanfeng Wang, Zhigang Wang

**Affiliations:** ^1^State Key Laboratory of Reproductive Regulation & Breeding of Grassland Livestock, School of Life Sciences, Inner Mongolia University, Hohhot, China; ^2^School of Life Sciences and Technology, Jining Normal University, Jining, China

**Keywords:** *Staphylococcus aureus*, amino acid, milk protein synthesis, bovine mammary epithelial cells, amino acid transporters, mTORC1

## Abstract

*Staphylococcus aureus* (*S. aureus*) is one of the main pathogens in cow mastitis, colonizing mammary tissues and being internalized into mammary epithelial cells, causing intracellular infection in the udder. Milk that is produced by cows that suffer from mastitis due to *S. aureus* is associated with decreased production and changes in protein composition. However, there is limited information on how mastitis-inducing bacteria affect raw milk, particularly with regard to protein content and protein composition. The main purpose of this work was to examine how *S. aureus* infection affects milk protein synthesis in bovine mammary epithelial cells (BMECs). BMECs were infected with *S. aureus*, and milk protein and amino acid levels were determined by ELISA after *S. aureus* invasion. The activity of mTORC1 signaling and the transcription factors NF-κB and STAT5 and the expression of the amino acid transporters SLC1A3 and SLC7A5 were measured by western blot or immunofluorescence and RT-qPCR. *S. aureus* was internalized by BMECs *in vitro*, and the internalized bacteria underwent intracellular proliferation. Eight hours after *S. aureus* invasion, milk proteins were downregulated, and the level of BMECs that absorbed Glu, Asp, and Leu from the culture medium and the exogenous amino acids induced β-casein synthesis declined. Further, the activity of mTORC1 signaling, NF-κB, and STAT5 was impaired, and *SLC1A3* and *SLC7A5* were downregulated. Eight hours of treatment with 100 nM rapamycin inhibited NF-κB and STAT5 activity, *SLC1A3* and *SLC7A5* expression, and milk protein synthesis in BMECs. Thus mTORC1 regulates the expression of *SLC1A3* and *SLC7A5* through NF-κB and STAT5. These findings constitute a model by which *S. aureus* infection suppresses milk protein synthesis by decreasing amino acids uptake in BMECs.

## Introduction

Bovine milk is an important source of nutrients, with diverse functions in humans, serving as a source of essential amino acids, providing immunological defense, and stimulating the absorption of nutrients (1, 2). Milk contains a wide array of proteins, which can be broadly classified into caseins and whey proteins. In bovine milk, caseins include αS1-, αS2-, β-, and κ-CN, and whey proteins include α-lactalbumin (α-LA), β-lactoglobulin (β-LG), serum albumin and immunoglobulins (3). Caseins are assembled in micelles, whereas whey proteins are soluble (4, 5). Casein is the principal protein in bovine milk, accounting for 75 to 80% of all proteins, and whey protein constitutes 15 to 20% (4, 6, 7). Increasing efforts have been undertaken to understand the regulatory mechanism of milk protein synthesis and improve protein concentrations in bovine milk.

The milk protein content in raw milk is governed by several factors, including the stage of lactation, nutrition supply, and disease (1). Mastitis, an intramammary type of inflammation, is a highly prevalent disease in dairy cows that causes significant economic losses in the bovine dairy industry. *Staphylococcus aureus* (*S. aureus*) is one of the main pathogens in bovine mastitis, with cell-bound properties on the surface that render the bacteria capable of adherence and invasion and secreted virulence factors that facilitate spread of the infection (8) and are often associated with cases of clinical mastitis (CM) and subclinical mastitis (SM) (8–12).

Milk that is produced by cows with mastitis due to *S. aureus* undergoes losses in production and changes in protein, that are abundance of cultured pathogens in milk-dependent (13, 14) or days in milk (DIM)-dependent (15). During mastitis, the protein composition is altered in the milk proteome (16, 17), wherein casein levels decrease in bovine milk, resulting in a lower yield, casein degradation, an imbalance between micellar and soluble casein, and changes in the stability and texture in fermented products (18–20). Moreover, the microbiological quality of raw milk is critical with regard to the quality of the final dairy product (19, 21). In recent years, researchers worldwide have conducted much work on improving the nutrient composition of milk to ensure milk quality and safety (1, 22, 23). However, there is limited information on how mastitis bacteria affects raw milk, particularly its protein content and protein composition.

Mechanistic (mammalian) target of rapamycin (mTOR) complex 1 (mTORC1) is the master regulator of cell growth and metabolism, responding to various environmental cues, including amino acids (24, 25). In addition to serving as the basic elements for protein synthesis, amino acids are irreplaceable for mTORC1 activation (26, 27), which recruits mTORC1 to the lysosomal surface, where it is activated (28–31). Data from the past several years have shown that several types of amino acids in lysosomes and the cytosol can be sensed by mTORC1 (32–35). mTORC1 is believed the most important regulator of protein synthesis, particularly translation, in all mammalian cells, through its downstream effectors, S6K1 and 4EBP1 (25, 36).

Bovine mammary epithelial cells (BMECs) synthesize and secrete milk and thus have been used widely as an *in vitro* cellular model to study the synthesis of milk protein in the udder of dairy cows (37–39). Recent work in mammary epithelial cells of dairy livestock has demonstrated the regulation of milk protein synthesis by mTORC1 (40–43). To synthesize milk protein, BMECs require the uptake of amino acids from extracellular fluid to improve the availability of intracellular amino acids, resulting in mTORC1 signaling activation (44–46); amino acid transporters are then used to concentrate amino acids in cells (46–48). Although mTORC1 and amino acid transporters are involved in milk protein synthesis (49–51), the effect of bacterial infection, particularly *S. aureus*, on mTORC1 signaling, amino acid uptake, and milk protein synthesis is unknown in BMECs.

To determine the mechanism by which intracellular infection by *S. aureus* affects milk protein synthesis in BMECs, we examined the uptake of amino acids; mTORC1 function in amino acid transporter expression; the expression of *CSN2* and its product, β-casein; *LALBA* and its protein, α-lactalbumin (α-LA); and *BLG* and its product, β-lactoglobulin (β-LG) in BMECs *in vitro* and measured the levels of β-caseins, α-LA, and β-LG in the cell culture medium. The purpose of this study was to develop a model by which intracellular infection by *S. aureus* suppresses milk protein synthesis, in which internalized bacteria inhibit mTORC1 activation and then prevent amino acid uptake in BMECs.

## Materials and Methods

### Ethics Statement

All experimental procedures with animals were conducted according to the guidelines for the care and use of experimental animals that have been established by the Inner Mongolia University Animal Care and Use Committee.

### Primary BMEC Culture

Primary BMECs were isolated and identified as described (52). Briefly, mammary tissue was obtained from Chinese Holstein cows after being slaughtered on a commercial cattle slaughter farm. After surgical removal of mammary tissue from the slaughtered cow, it was placed in sterile, ice-cold phosphate-buffered saline (PBS) that was supplemented with 300 U/mL penicillin G and 100 mg/mL streptomycin (Sigma-Aldrich, Inc., USA) and transported immediately to the laboratory. Purified primary BMECs were isolated and maintained in DMEM/F12 medium (Hyclone Laboratories, Inc., Logan, UT, USA) that contained 10% fetal bovine serum. Cells were cultured in 25 cm^2^ tissue culture flasks at 37°C in humidified air with 5% CO_2_. P2 to P4 BMECs that were in the logarithmic growth phase were used for all experimental assays.

### Reagents and Antibodies

Glu (Cat# G8415), Asp (Cat# A7219), and Leu (Cat# L8912) were purchased from Sigma-Aldrich, Inc. (St. Louis, MO, USA). β-casein (Cat# EIA06975Bo) was purchased from Wuhan Xinqidi (Wuhan Xinqidi Biological Technology Co. Ltd. Wuhan, China). Rapamycin (Cat# 53123-88-9) was purchased from Gene Operation (Gene Operation, Ann Arbor, MI, USA). Rapamycin was dissolved in ethanol (Sigma-Aldrich, Inc., USA) to a stock concentration of 50 mg/mL, stored at −20°C, and diluted to the appropriate final concentration with culture medium before use. The concentration of ethanol in the final solution did not exceed 0.5% (v/v) in any experiment. β-mercaptoethanol (Cat# M8211) was obtained from Solarbio (Solarbio Science & Technology, Co., Ltd. Beijing, China). Dil (Cat# KGMP002) was purchased from KeyGEN (KeyGEN BioTECH, Co., Ltd. Jiangsu, China). DAPI (Cat# C1005) and Hoechst 33342 Staining Kit (Cat# C1022) were acquired from Beyotime (Beyotime Biotechnology, Co., Ltd. Shanghai, China). Annexin V-FITC/PI Cell Apoptosis Detection Kit (Cat# FA101) was purchased from TransGen (TransGen Biotech Co. Ltd. Beijing, China). Alexa Fluor® 594 Phalloidin (Cat# A12381) was purchased from Invitrogen (Invitrogen, Carlsbad, New Mexico, USA). 5(6)-carboxyfluorescein diacetate N-succinimidyl ester (CFSE) were purchased from Sigma (Sigma-Aldrich, Inc. St. Louis, MO, USA).

Anti-4EBP1 (Cat# ab2606), anti-p-mTOR (Ser2448) (Cat# ab109268), anti-mTOR (Cat# ab32028), goat anti-rabbit (Cat# ab136817), and goat anti-mouse (Cat# ab205719) were purchased from Abcam (Abcam plc 330 Cambridge Science Park, Cambridge, UK). Anti-p-S6 (Ser240/244) (Cat# 5364s), anti-p-4EBP1 (Thr37/46) (Cat# 2855s), anti-p-NF-κB p65 (Ser536) (Cat# 3033), anti-NF-κB p65 (Cat# 8242), and anti-p-STAT5 (Tyr694) (Cat# 4322) were purchased from Cell Signaling Technology (Cell Signaling Technology, Inc., Beverley, MA, USA). Anti-S6 (Cat# sc-74459) was purchased from Santa Cruz Biotechnology (Santa Cruz Biotechnology, Inc., 10410 Finnell Street Dallas, Texas 75220 USA). Anti-STAT5A (Cat# 13179-1-AP), anti-SLC1A3 (Cat# 20785-1-AP), anti-SLC7A5 (Cat# 13752-1-AP) and anti-Caspase 3 (Cat# 66470-2-Ig) were purchased from Proteintech (Proteintech Group, Inc., 5500 Pearl Street, Suite 400 Rosemont, IL 60018, USA). Goat FITC-conjugated anti-rabbit IgG (Cat# 115-095-003) and FITC-conjugated anti-mouse IgG (Cat# 115-095-146) were purchased from Jackson (Jackson ImmunoResearch Laboratories, Inc., West Grove, PA, USA). Anti-β-actin (Cat# A5441) was purchased from Sigma (Sigma-Aldrich, Inc., St. Louis, MO, USA).

### Spread Plate Method

BMECs were infected with *S. aureus* (ATCC 27543) for 2 h at an MOI of 30, and the extracellular bacteria were killed and lysed with antibiotics and lysozyme. Intracellularly infected cell cultures were continued and maintained in medium for 2, 4, and 8 h. The cells were lysed, and the number of intracellular bacteria was determined by spread plate method.

### Bacterial Staining

BMECs were seeded on a slide and incubated overnight. Bacteria (*S*. *aureus*) were washed with PBS and then incubated with CFSE [5 (6)-carboxyfluorescein diacetate N-succinimidyl ester] at 4°C for 15 min. The stained bacteria were centrifuged for 10 min at 3,000 × g at 4°C 3 times. Cells were infected by the stained bacteria at an MOI of 30 for 2 h, washed three times with PBS, and fixed with 4% paraformaldehyde for 20 min. After being treated with 1% Triton X-100 for 5 min, the cells were stained with Alexa Fluor® 594 Phalloidin for 1 h in the dark, washed three times with PBS, and counterstained with 100 μl DAPI for 3 min to assess the nuclear morphology. Finally, the slide was mounted with glycerin for examination under a laser scanning confocal microscope (NIKON A1R, Nikon Corp., Tokyo, Japan).

### TEM

BMECs were infected with bacteria (*S. aureus*) for 2 h at an MOI of 30, and the extracellular bacteria were killed and lysed with antibiotics and lysozyme for 2 h. The infected cells were washed three times with PBS, centrifuged for 10 min at 3,000 × g at 4°C, and fixed with 2.5% glutaraldehyde overnight, the precipitation was wrapped in the 1% agarose. Agarose blocks with samples avoid light post fixed with 1% OsO_4_ in 0.1 M PB (pH 7.4) for 2 h at room temperature. The tissues were sequentially fed with 30%-50%-70%-80%-95%-100%-100% alcohol and dehydrated for 20 min each time, 100% acetone twice, 15 min each time. Resin penetration and embedding as followed: Acetone, EMBed 812 = 1:1 for 2–4 h at 37°C; Acetone, EMBed 812 = 1:2 overnight at 37°C; pure EMBed 812 for 5–8 h at 37°C; Pouring the pure EMBed 812 into the embedding models and insert the tissues into the pure EMBed 812, and then keep in 37°C overnight. The embedding models with resin and samples were moved into 65°C to polymerize for more than 48 h. The resin blocks were cut to 60–80 nm thin on the ultra microtome, and the tissues were fished out onto the 150 meshes cuprum grids with formvar film, and the 2% uranium acetate saturated alcohol solution avoid light staining for 8 min and then rinsed in 70% ethanol for three times. 2.6% Lead citrate avoid CO_2_ staining for 8 min. After dried by the filer paper, the cuprum grids were put into the grids board and dried overnight at room temperature. Finally, the samples were examined by TEM (Hitachi HT7700, Hitachi, Ltd., Tokyo, Japan) to detect intracellular bacteria.

### ELISA

BMECs were seeded into 6-well plates, incubated until 80% confluence, and treated with the indicated conditions, including infection with *S*. *aureus*; serum and amino acid starvation, followed by amino acid stimulation and rapamycin.

To examine how *S. aureus* invasion suppresses milk protein synthesis, BMECs were infected with *S*. *aureus* for 2 h, and the extracellular bacteria were killed and lysed with antibiotics and lysozyme. The intracellularly infected cells were continued in culture and maintained in medium for 8 h. Cell culture supernatants were collected to measure extracellular β-casein, α-lactalbumin, and β-lactoglobulin using ELISA kits (Wuhan Xinqidi Biological Technology Co. Ltd. Wuhan, China) per the manufacturer's instructions. Intracellularly infected cells were harvested with trypsin and centrifuged to remove the supernatant, and cell lysates were prepared through five freeze-thaw cycles. The total protein concentration of the control and treatment groups was standardized by adjusting the volume of the protein lysate. An equal volume of each total protein lysate was analyzed for β-casein, α-lactalbumin, and β-lactoglobulin by ELISA.

To determine how rapamycin treatment decreases milk protein synthesis, BMECs were treated with 100 nM rapamycin for 8 h, the cell culture supernatants were collected, the cells were harvested, and cell lysates were prepared and standardized. The levels of extracellular and intracellular β-casein, α-lactalbumin, and β-lactoglobulin were analyzed by ELISA.

To examine how exogenous amino acids induce casein synthesis, BMECs were serum-starved for 16 h, amino acid-starved for 1 h, and then stimulated with amino acids for 1 h. Control and treated cells were harvested, and cell lysates were prepared and standardized. The level of intracellular β-casein was analyzed by ELISA.

To study the suppression of amino acid uptake by *S*. *aureus*, BMECs were serum-starved for 16 h, amino acid-starved for 1 h, stimulated with amino acids for 1 h, and infected intracellularly with *S*. *aureus* for 8 h. Cell culture supernatants were collected to measure Glu, Asp, and Leu using ELISA kits (Wuhan Xinqidi Biological Technology Co. Ltd. Wuhan, China).

To examine how *S. aureus* invasion suppresses amino acid induced-casein synthesis, four groups of BMECs were compared: control, amino acid induction (Glu, Asp, Leu), *S. aureus* invasion (8 h), and amino acid stimulation with bacterial infection. BMECs were serum starved for 16 h, amino acid-starved for 1 h, stimulated with amino acids for 1 h, and infected intracellularly with *S*. *aureus* for 8 h. Control and treated cells were harvested, and cell lysates were prepared and standardized. Intracellular β-casein was analyzed by ELISA.

To determine the contents of alpha-hemolysin (Hlα) and Plasmin, BMECs were infected with *S. aureus* for 2 h, and the extracellular bacteria were killed and lysed with antibiotics and lysozyme. The contents of α-hemolysin (Hla), and Plasmin in cell medium and in cells were measured after infection 8 h using ELISA kits (Wuhan Xinqidi Biological Technology Co. Ltd. Wuhan, China). To determine whether β-casein was directly degraded by *S. aureus* in culture medium, 1.5 × 10^3^ CFU/mL *S. aureus* were inoculated into DMEM/F12 medium which β-casein was dissolved to the final concentration of 1 μg/mL, and maintained in 37°C. The content of β-casein was determined after 8 h.

Absorbance at 450 and 630 nm was read on a Varioskan Flash Multimode Reader (Thermo Fisher Scientific, Pittsburgh, PA, USA). All measurements were performed in triplicate, and the mean value of the 3 independent measurements was used for statistical analysis.

### Western Blot

Western blot was used to measure the indicated proteins and phosphorylated proteins as described (53). Briefly, BMECs were managed as four groups, i.e., control cells (uninfected cells), cells were infected by *S. aureus* 2, 4, and 8 h, respectively. Four groups of cells were culture in medium simultaneously, and then three infected groups were inoculated with *S. aureus* at different time points and continued in co-culture. Finally, the cells were harvested with trypsin at the same time. The harvested BMECs were washed with cold PBS, and lysed in cell lysis buffer. The lysis buffer comprised 50 mM Tris (pH 7.4), 150 mM NaCl, 1% Triton X-100, 1% sodium deoxycholate, 0.1% SDS, PMSF, and phosphatase inhibitors. Equal amounts (40 μg) of protein were electrophoresed on 10% (w/v) sodium dodecyl sulfate-polyacrylamide gels, transferred to polyvinylidene fluoride membranes, and incubated with the primary antibody. Peroxidase-conjugated secondary antibody and enhanced chemiluminescence (ECL) reagent were used to detect the signals with the Western Blotting System (GE Healthcare Bio-Sciences, Pittsburgh, PA, USA). The bands were quantified on a Gel-Pro Analyzer 4.0 (Media Cybernetics, USA).

### RT-qPCR

RT-qPCR was performed to measure *EAAT1/GLAST/SLC1A3 and LAT1/SLC7A5* in BMECs in the treatment and control groups. Cells were infected with *S. aureus* for 2, 4, and 8 h or treated with 100 nM rapamycin for 8 h, and total RNA was extracted from untreated and treated cells. Total RNA was prepared with RNAiso Plus per the manufacturer's instructions (9109, TaKaRa Co. Ltd., Dalian, China). Briefly, the cells were washed with PBS and lysed in RNAiso Plus, and chloroform was added to the cell lysates for homogenization; the top aqueous layer was transferred to a new tube after centrifugation, and isopropanol was added to the supernatant and mixed well. Total RNA was precipitated by centrifugation, and the pellet was dissolved in RNase-free water.

mRNA was reverse-transcribed with oligo (dT)_12−18_ primer using the AMV first Strand cDNA Synthesis Kit (Takara Co. Ltd., China). cDNA sequences were amplified with the primers in Supplementary Table S1. The KAPA SYBP FAST qPCR Kit Optimized for LightCycler 480 (KAPA, Inc., Boston, MA, USA) was used for the PCR with the primers (Supplementary Table S1), according to the manufacturer's instructions. The program comprised an initial denaturation step at 95°C for 5 min; 40 cycles of 95°C for 5 s, 54°C for 30 s, and 72°C for 20 s; and a final extension of 72°C for 10 min. Three technical replicates were run in each experiment. 2-ΔΔCT values were calculated to determine expression levels, and the qPCR results were compared by student's *t*-test between untreated and treated groups. Three independent experiments were performed.

### Immunofluorescence

Cells were seeded onto a slide, incubated overnight, and infected with *S. aureus* for 2, 4, and 8 h. After being washed with PBS and fixed with 4% paraformaldehyde for 15 min, the cells were blocked with 1% BSA for 1 h. Then, the cells were incubated with primary antibodies against SLC1A3 and SLC7A5, p-STAT5 and p-NF-κB p65 at 4°C overnight and FITC-labeled goat anti-rabbit IgG for 1 h at room temperature. DAPI was used to stain the nucleus. Finally, the slide was mounted with glycerin and examined under a laser scanning confocal microscope (NIKON A1R, Nikon Corp., Tokyo, Japan).

### Adhesion Assays

Adhesion assay of *S. aureus* was achieved in two phases. First, BMECs were infected with *S. aureus* at MOI 30 for 30, 60, and 90 min, respectively. End of infection, BMECs were continued and maintained for 8 h in medium with antibiotics and lysozyme. After incubation, BMECs were harvested with trypsin and washed softly three times with PBS to remove extracellular dead bacteria, and then lysed using lysis buffer. The number of intracellular bacteria was determined by bacterial colony count. Second, BMECs were infected with *S. aureus* for 30 min at MOI 30, and then the cells were cultured for 8 h in a medium with antibiotics and lysozyme. After incubation, BMECs were harvested with trypsin and washed softly three times with PBS to remove non-adherent bacteria, remaining bacteria considered to be adherent but not internalized in cells, and then lysed using lysis buffer. The bacteria were evaluated by bacterial colony count, which were considered as adherent bacteria.

### Apoptosis Analysis

BMECs were infected with *S. aureus* at MOI 30 for 2 h, then the antibiotics and lysozyme were used to kill and lyse the extracellular bacteria. The intracellularly infected cells were continued in culture and maintained in medium for 8 h, and then the apoptosis were assessed with the Hoechst 33342 Staining Kit and FITC Annexin V Apoptosis Detection Kit according to the manufacturer's instructions, respectively. Following treatment, cells were stained with Hoechst for 5 min and washed with PBS, followed by observation under a fluorescence microscope (Observer A1, Zeiss, Oberkochen, Germany). For the flow cytometry assay, cells were collected after treatment, and washed with PBS, and then stained with FITC-Annexin V and PI. Cells were subsequently analyzed using flow cytometry (Cytoflex, Beckman, CA, USA).

### Statistical Analysis

Statistical analyses were conducted using SPSS PASW Statistics for Windows, v18.0 (SPSS Inc.: Chicago, IL, USA). Data were analyzed using standard parametric statistics and one-way ANOVA, followed by Tukey's method. Data are expressed as mean ± SD. The results are presented as the average of at least 3 independent experiments. Western blot results were quantified on a Gel-Pro Analyzer 4.0 (Media Cybernetics, USA). Statistical significance was accepted when *p* ≤ 0.05.

## Results

### *Staphylococcus aureus* Invasion Suppresses Milk Protein Synthesis and Prevents Uptake of Exogenous Amino Acids in BMECs

In order to confirm *S. aureus* can be internalized by BMECs, we infected BMECs with *S. aureus* for 2 h at MOI 30 and then killed and lysed the extracellular bacteria with antibiotics and lysozyme. The infected cells were maintained in medium for 2-8 h, and the bacteria were evaluated intracellularly and extracellularly by bacterial colony count. The results showed that 1.5 × 10^3^ CFU/mL − 5.1 × 10^3^ CFU/mL were counted in whole BMEC lysates, whereas extracellular bacteria were not found in the culture medium (Supplementary Table S2), indicating that intracellular *S. aureus* proliferated. Further, to confirm *S. aureus* invasion of BMECs, we stained *S. aureus* with fluorescent dye and observed the bacteria under a laser scanning confocal microscope (LSCM) (Supplementary Figure S1A). *S. aureus* was also found in cytosolic vacuoles in BMECs by TEM (Supplementary Figure S1B). These results indicate that *S. aureus* was internalized by BMECs *in vitro*.

**Figure 1:**
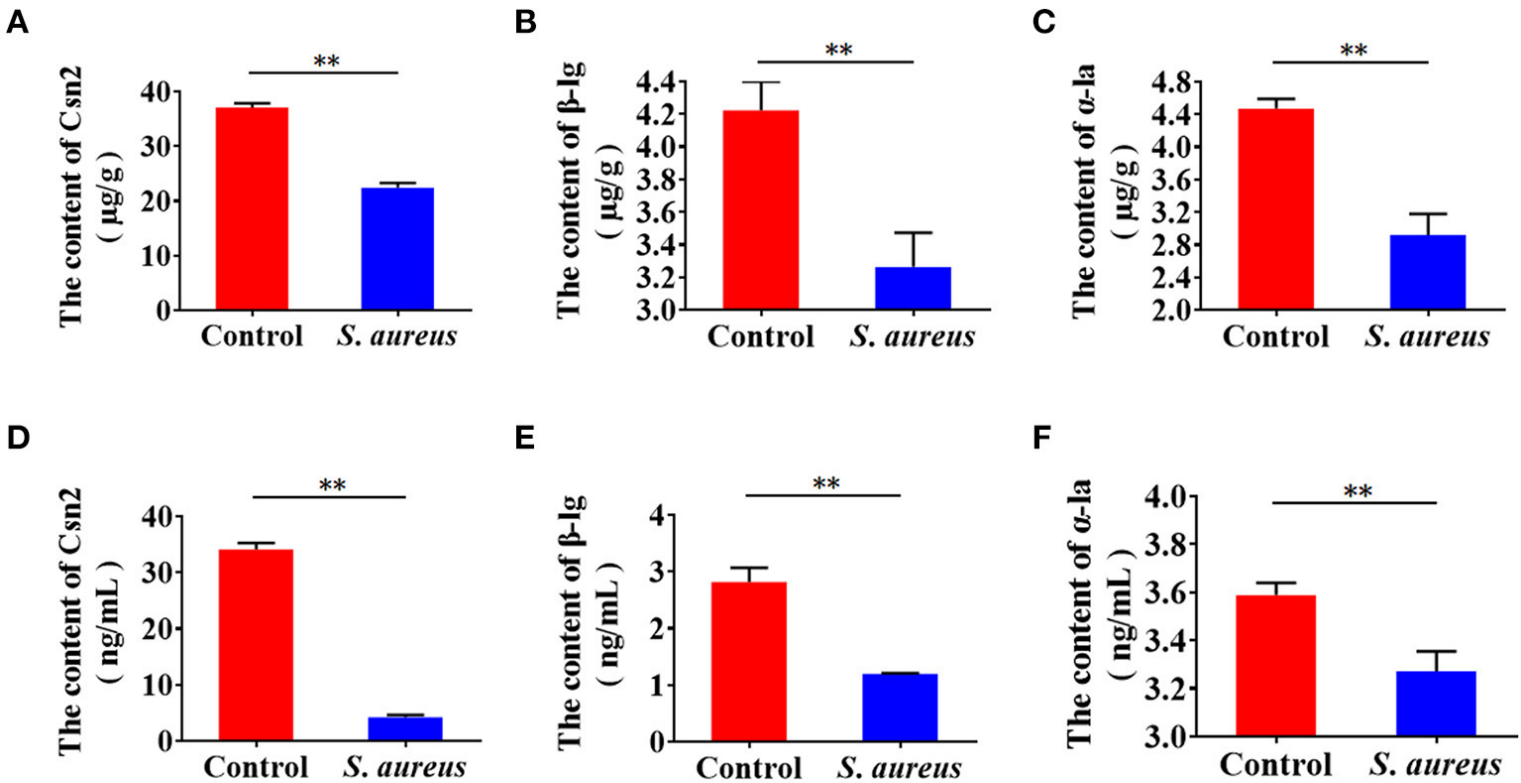
*Staphylococcus aureus* invasion suppresses milk protein synthesis and secretion in BMECs 8 h after infection. **(A–C)** Levels of intracellular Csn2 (β-casein), β-lg, and α-la. **(D–F)** Levels of Csn2 (β-casein), β-lg and α-la in cell culture medium. ^**^p < 0.01. *n* = 3 independent experiments.

To examine whether *S. aureus* invasion suppresses the synthesis of milk protein in BMECs, cells were infected with *S. aureus* at 30 MOI for 2 h, and extracellular bacteria were then killed and lysed with antibiotics and lysozyme. The infected cells were maintained in medium for 8 h, and β-casein, α-lactalbumin, and β-lactoglobulin were determined by ELISA. The levels of β-casein, α-lactalbumin and β-lactoglobulin decreased intracellularly (Figures 1A–C) and in medium (Figures 1D–F), indicating that *S. aureus* invasion inhibits the synthesis of milk protein in BMECs.

Considering milk protein can be degraded by endogenous protease or bacterial enzymes, to eliminate the possibility of milk proteins were degraded by these enzymes, we first determined the level of endogenous protease Plasmin by ELISA in *S. aureus* infected cells, and found that the level of Plasmin was not increased in the culture medium and in cells of the *S. aureus*-infected cells, compared to control (Supplementary Figure S2A). Next, to determine whether β-casein was directly degraded by *S. aureus* during infection of 8 h, we simulated the conditions under which *S. aureus* infected BMECs, i.e., 1.5 × 10^3^ CFU/mL *S. aureus* (Supplementary Table S2) were inoculated into DMEM/F12 medium which β-casein, and the content of β-casein was determined by ELISA after 8 h. Comparing to the control, the level of β-casein did not show significant decline in infected group (Supplementary Figure S2B). These data indicate that the decrease in milk protein content was caused by intracellular infection of *S. aureus*, rather than by both endogenous and bacterial enzymes. Then, to eliminate the possibility of apoptosis induced by *S. aureus* leading to the decrease of milk protein, we examined apoptosis 8 h after *S. aureus* infection, and the results showed that no apoptosis was found in BMECs (Supplementary Figures S3A–C), suggesting that the decrease of milk protein was not caused by apoptosis.

**Figure 2:**
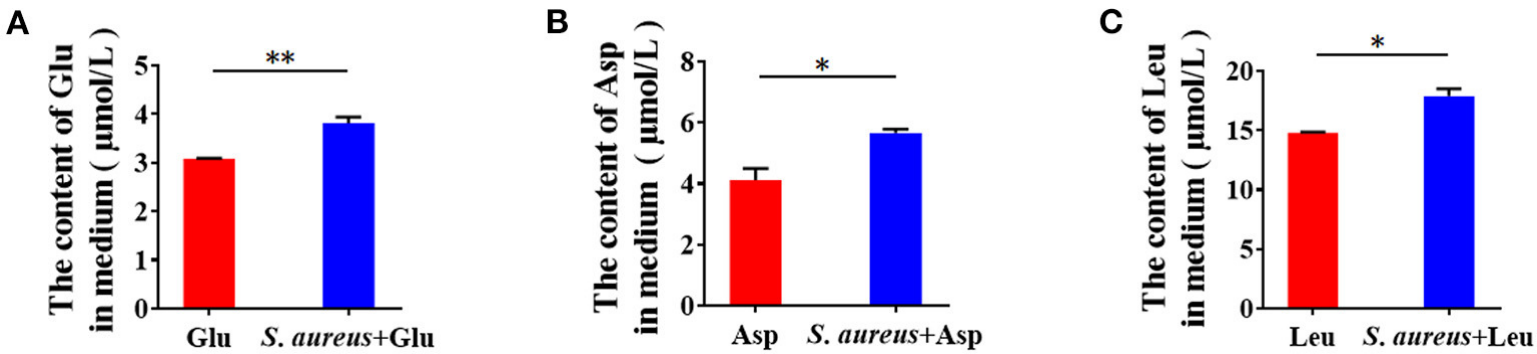
*Staphylococcus aureus* invasion prevents amino acid uptake in BMECs 8 h after infection. **(A–C)** Glu **(A)**, Asp **(B)**, and Leu **(C)** content in medium. *p < 0.05; **p < 0.01. *n* = 3 independent experiments.

Epithelial cells are the central component of bovine mammary alveoli, which produce milk during lactation. Mammary epithelial cells are considered to derive amino acids from blood to synthesize milk proteins. Thus, we tested whether *S. aureus* invasion prevents cells from absorbing amino acids from the culture medium. Control and *S. aureus*-infected BMECs were subjected to serum and amino acid starvation, after which Glu, Asp, and Leu were added to the medium and measured by ELISA. The levels of Glu, Asp, and Leu in the medium of *S. aureus*-infected cells was significantly higher than that in the control (Figures 2A–C), indicating that *S. aureus* prevents BMECs from taking up amino acids from the culture medium.

### Exogenous Amino Acids Induce Casein Synthesis and mTORC1 Activation

**Figure 3:**
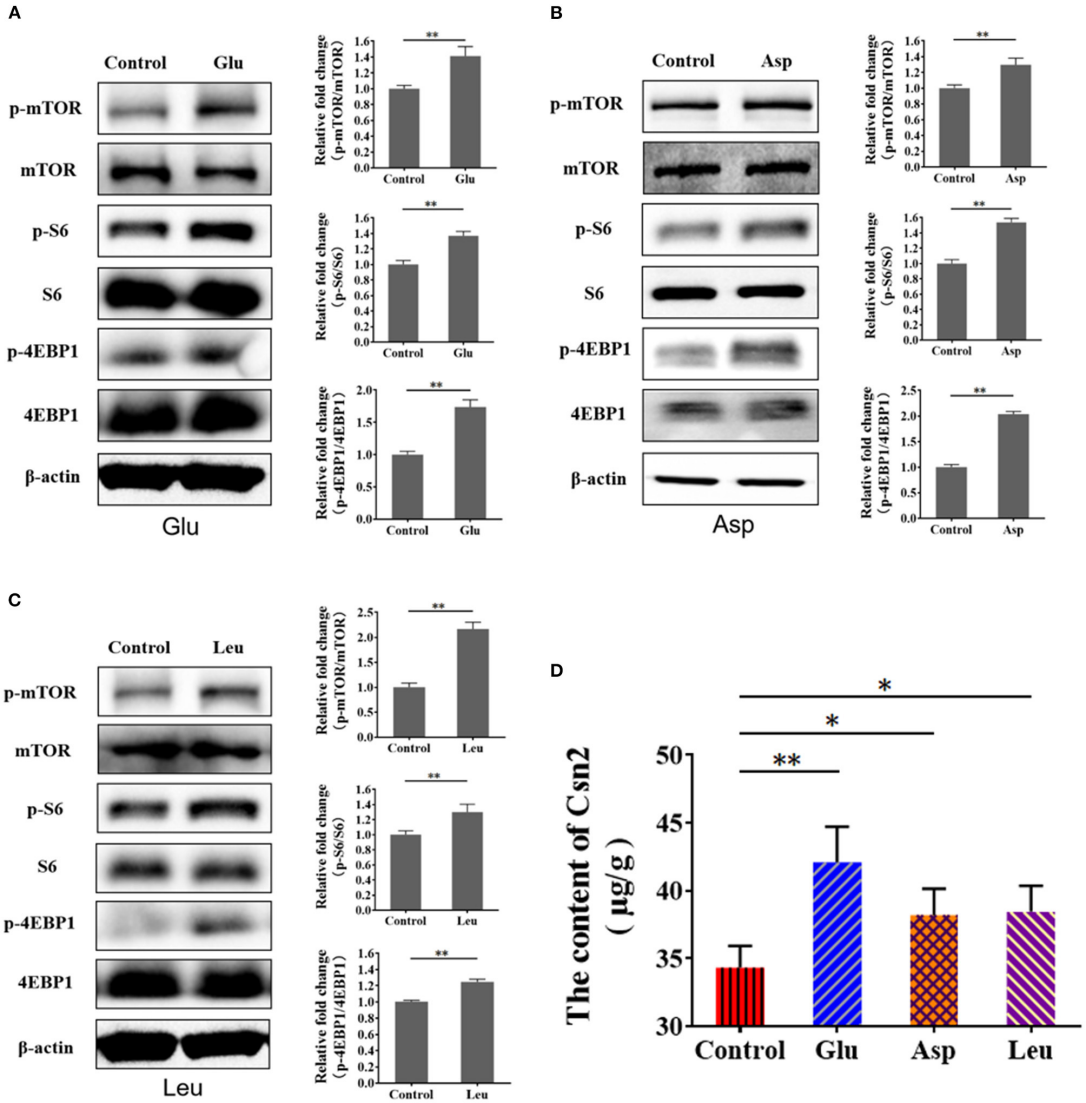
Exogenous amino acid promotes mTORC1 activation and CSN2 (β-casein) synthesis. **(A–C)** Western blot of mTORC1 activation after BMEC stimulation with Glu **(A)**, Asp **(B)**, and Leu **(C)**. Phosphorylation of mTOR, S6, and 4EBP1. **(D)** Intracellular Csn2 (β-casein). The resolved bands were quantified using Gel-Pro Analyzer 4.0 (Media Cybernetics, Inc., Rockville, MD, USA). *p < 0.05; **p < 0.01. *n* = 3 independent experiments.

Amino acids initiate mTORC1 signaling to promote protein synthesis. Thus, we speculated that mTORC1 activation and milk protein synthesis are stimulated by exogenous amino acids in BMECs. We treated serum- and amino acid-starved cells with Glu, Asp, and Leu and measured mTORC1 activation and β-casein (Csn 2) in BMECs. The results showed that mTORC1 activation (Figures 3A–C) and β-casein (Figure 3D) were increased in BMECs, demonstrating that exogenous amino acids initiate mTORC1 activation and induce β-casein (Csn 2) synthesis.

### *Staphylococcus aureus* Invasion Suppresses Amino Acid Induced-Casein Synthesis

**Figure 4:**
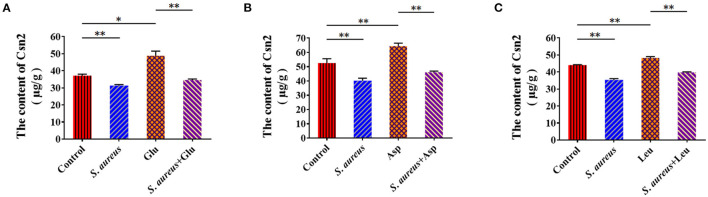
*Staphylococcus aureus* invasion suppresses amino acid induced-casein synthesis. Intracellular Csn2 (β-casein) content **(A)** Glu, **(B)** Asp, and **(C)** Leu. *p < 0.05; **p < 0.01. *n* = 3 independent experiments.

To characterize the suppression of amino acid-induced casein synthesis by *S. aureus*, β-casein was measured in four groups of BMECs: control, amino acid-treated (Glu, Asp, Leu), *S. aureus* invasion, and *S. aureus* invasion with amino acids (Glu, Asp, Leu). Exogenous amino acids significantly increased β-casein concentrations, an effect that *S. aureus* infection mitigated (Figures 4A–C). These data indicate that *S. aureus* invasion suppresses amino acid-induced casein synthesis.

### *Staphylococcus aureus* Invasion Downregulates Amino Acid Transporter Genes and the Phosphorylation of NF-κB, STAT5, mTOR, and S6

**Figure 5:**
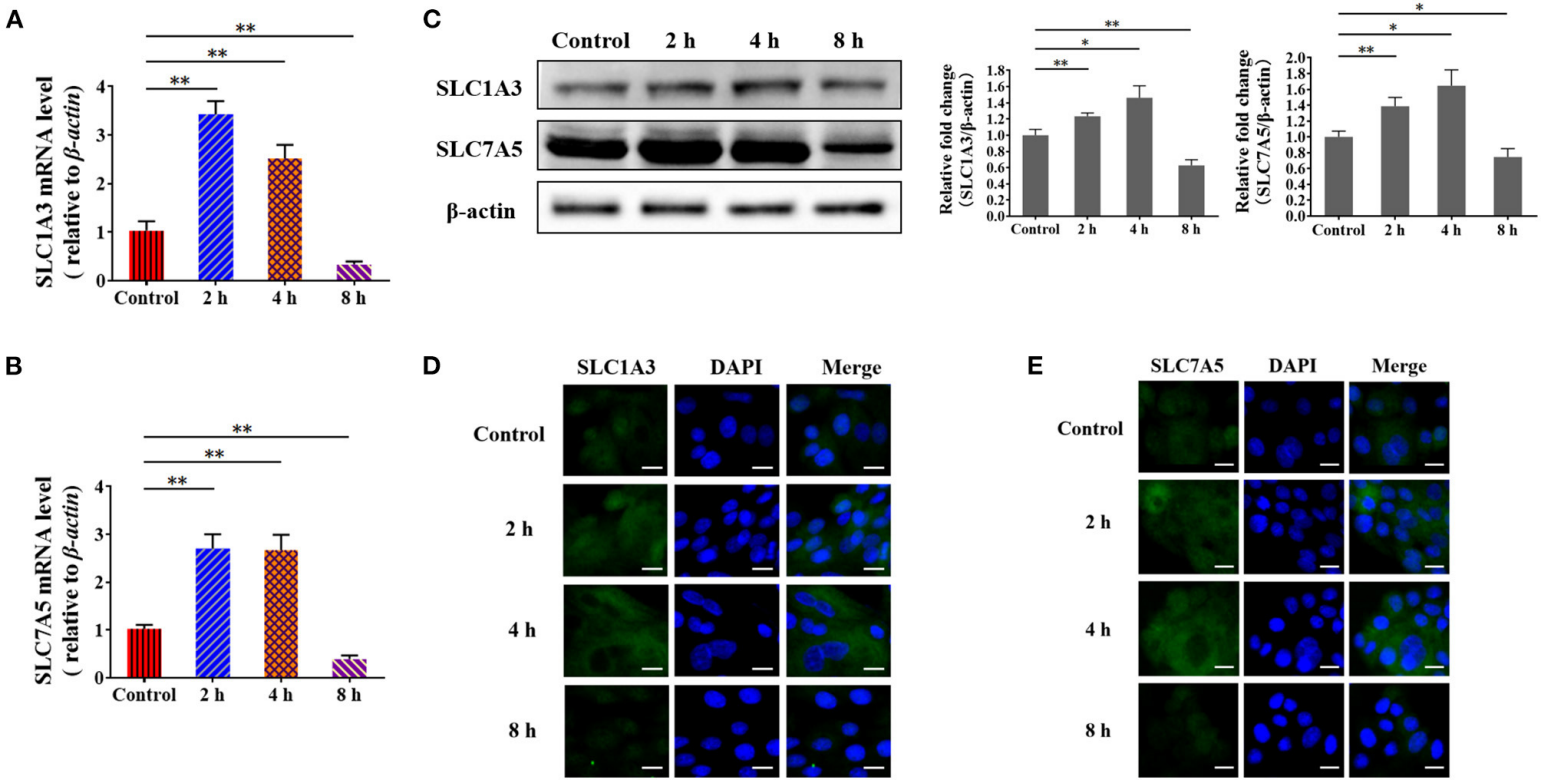
*Staphylococcus aureus* infection impairs the expression of amino acid transporter genes at 8 h. **(A,B)** mRNA levels of *SLC1A3* **(A)** and *SLC7A5* **(B)** in *S. aureus*-infected BMECs. **(C)** Protein levels of SLC1A3 and SLC7A5 in *S. aureus*-infected BMECs. **(D,E)** Immunofluorescence assay of SLC1A3 **(D)** and SLC7A5 **(E)** in infected BMECs and control. Representative confocal microscopy images of SLC1A3 and SLC7A5 (Green) in cells that were co-stained with DAPI (blue). Scale bars represent 20 μm. The resolved bands were quantified using Gel-Pro Analyzer 4.0 (Media Cybernetics, Inc., Rockville, MD, USA). *p < 0.05; **p < 0.01. *n* = 3 independent experiments.

To validate the underlying mechanism by which *S. aureus* invasion prevents amino acid uptake and amino acid-induced casein synthesis in BMECs, we examined the expression of *SLC1A3*(*EAAT1/GLAST*) and *SLC7A5*(*LAT1*) by RT-qPCR. The mRNA levels of *SLC1A3* and *SLC7A5* increased at 2 and 4 h and declined at 8 h in *S. aureus*-invaded BMECs (Figures 5A,B). SLC1A3 and SLC7A5 were detected by western blot and immunofluorescence, following the same trend as the mRNA levels in *S. aureus*-infected BMECs (Figures 5C–E). These results suggest that *S. aureus* invasion impairs the expression of amino acid transporter genes at the mRNA and protein levels in BMECs.

**Figure 6:**
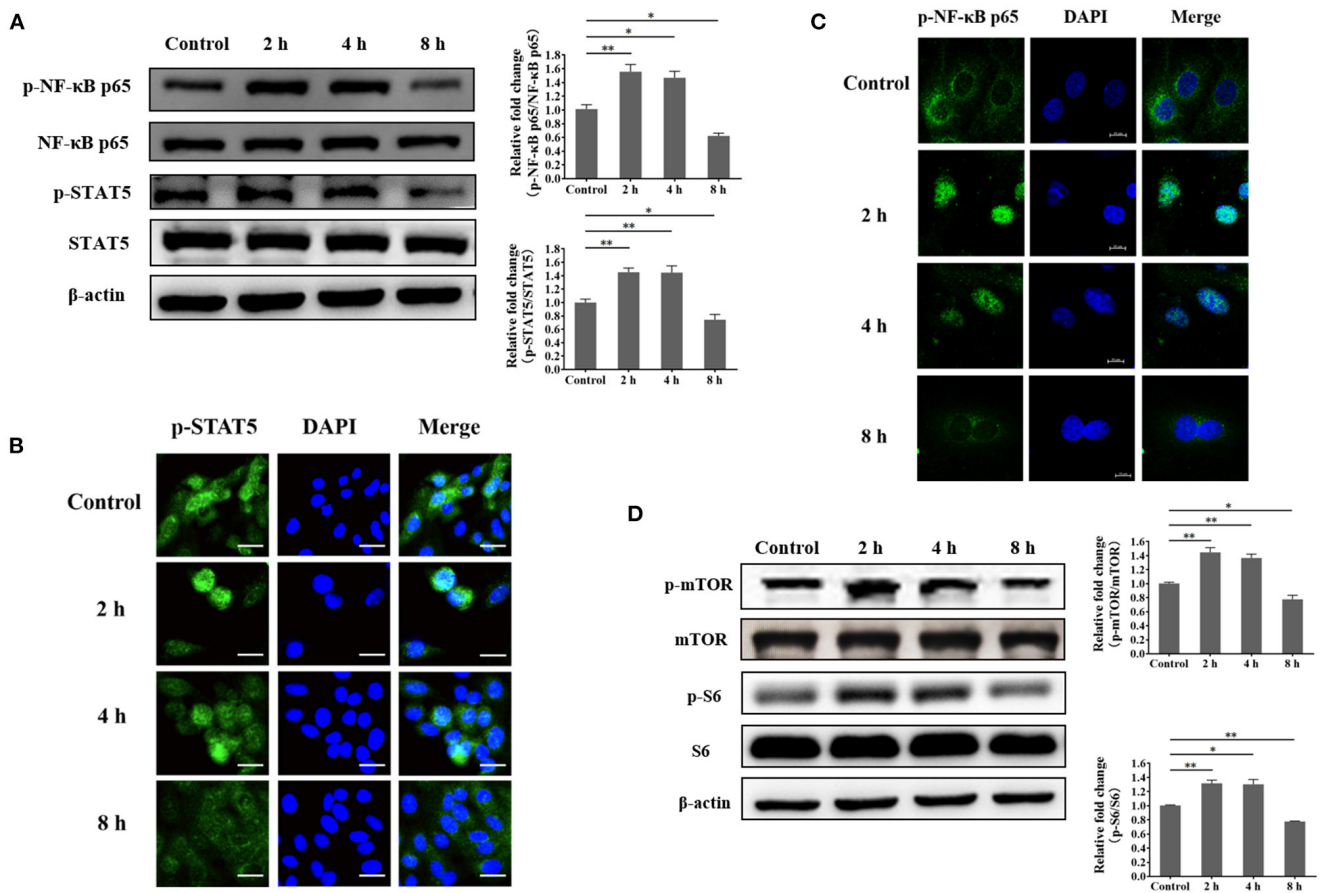
*Staphylococcus aureus* infection suppresses the phosphorylation of NF-κB p65, STAT5, mTOR, and S6 at 8 h. **(A)** Phosphorylation of NF-κB p65 and STAT5 in *S. aureus*-invaded BMECs. **(B)** Nuclear localization of phosphorylated STAT5 in *S. aureus*-invaded BMECs. Representative confocal microscopy images of the level of p-STAT5 (Green) in cells that were co-stained with DAPI (blue). Scale bars represent 20 μm. **(C)** Nuclear localization of phosphorylated NF-κB p65 in *S. aureus*-invaded BMECs. Representative confocal microscopy images of the level of p-NF-κB p65 (Green) in cells that were co-stained with DAPI (blue). Scale bars represent 10 μm. **(D)** mTORC1 signaling in *S. aureus*-invaded BMECs. The resolved bands were quantified using Gel-Pro Analyzer 4.0 (Media Cybernetics, Inc., Rockville, MD, USA). *p < 0.05; **p < 0.01. *n* = 3 independent experiments.

Based on the findings, we speculated that certain transcription factors that are related to these genes are also impaired in *S. aureus*-invaded BMECs. To identify transcription factors for *SLC1A3* and *SLC7A5*, we performed a bioinformatic analysis, which predicted NF-κB and STAT5 (Supplementary Figure S4, S5). Further, we examined the phosphorylation of NF-κB and STAT5 and nuclear localization by western blot and immunofluorescence. We found that the phosphorylation of NF-κB and STAT5 was reduced, and the nuclear translocation of phosphorylated NF-κB p65 and STAT5 was also attenuated at 8 h after bacterial infection (Figures 6A–C). These data suggest that *SLC1A3* and *SLC7A5* expression is directed by NF-κB and STAT5 in BMECs. The experiments above demonstrate that exogenous amino acids induce mTORC1 signaling and that *S. aureus* invasion prevents the uptake of exogenous amino acids. Thus, we speculated that *S. aureus* invasion decreases the activity of mTORC1 in BMECs. In *S. aureus-*infected BMECs, the phosphorylation of mTOR and S6 fell 8 h after invasion (Figure 6D), indicating that mTORC1 is involved in the expression of *SLC1A3* and *SLC7A5*.

Considering non-invasive *S. aureus* is also very important in mastitis, to eliminate the possibility of *S. aureus* adhesion to affect the activity of mTORC1, and NF-κB and STAT5, and the expression of SLC1A3 and SLC7A5, we first examined adhesion of *S. aureus* to BMECs, and found that the bacteria only adhered to BMECs but failed to internalize within 30 min. Next, BMECs were infected with *S. aureus* for 30 min, and then the cells were cultured for 8 h in a medium with antibiotics and lysozyme. The content of β-casein, α-lactalbumin, and β-lactoglobulin in cell culture medium was determined by ELISA, and the expression of the targeting proteins by Western blot. The results showed that there was no significant difference between the bacterial adhesion group and the control group (Supplementary Figures S6A–C), indicating that *S. aureus* adhesion has no effect on the milk protein synthesis, mTORC1 signaling, the activity of NF-κB p65 and STAT5, and the expression of SLC1A3 and SLC7A5. Further, to eliminate the possibility of *S. aureus* toxin effect on milk protein synthesis, alpha-hemolysin (Hlα), which is the most abundant toxin in *S. aureus*, was determined by ELISA after infection 8 h. The results showed that α-hemolysin was not detectable both in cell medium and cells (Supplementary Figure S7), suggesting that toxins were not produced by *S. aureus* within 8 h.

### mTORC1 Regulates the Expression of *SLC1A3* and *SLC7A5* Through NF-κB and STAT5 in BMECs

**Figure 7:**
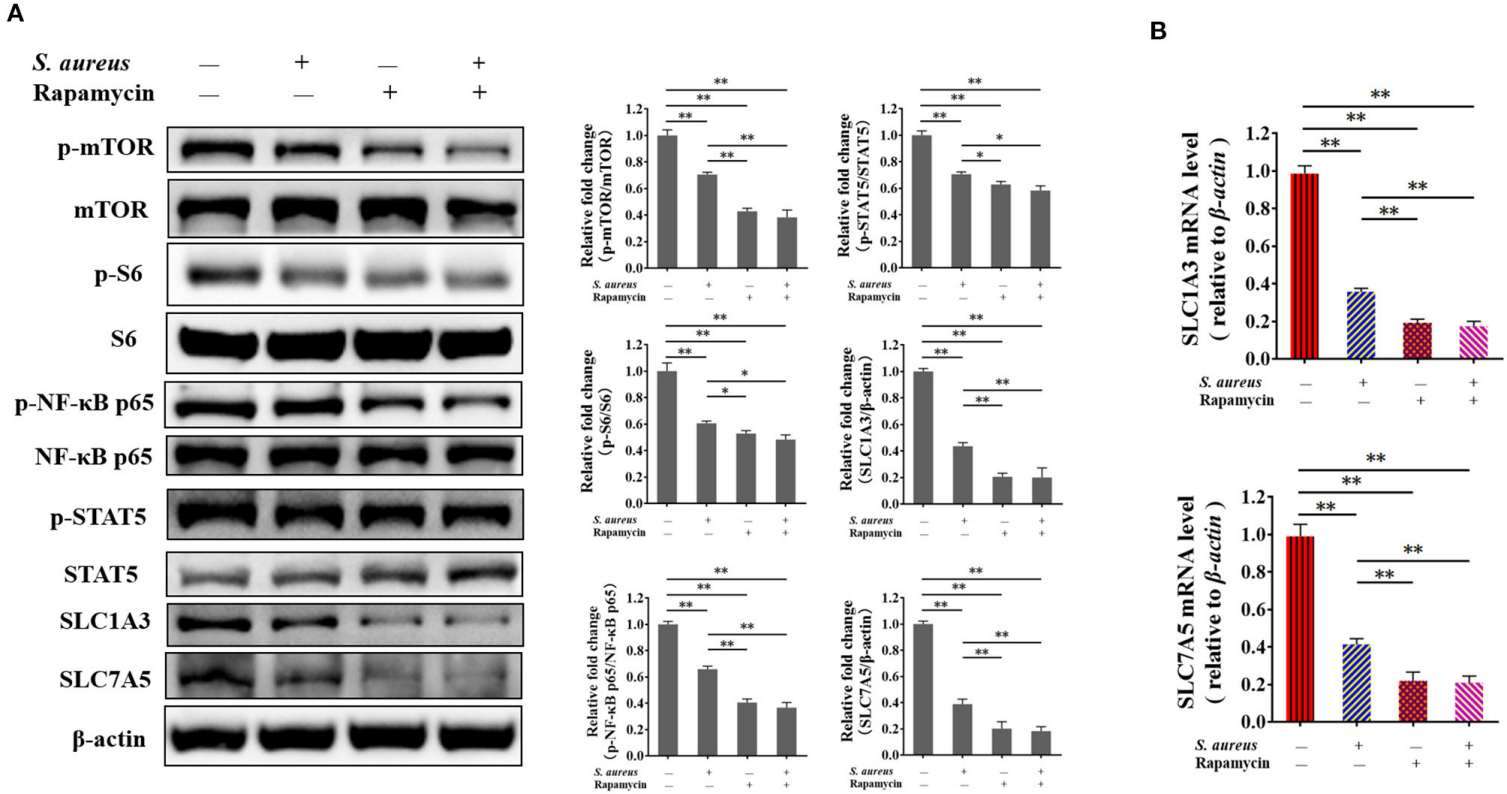
Inhibition of mTORC1 by *S. aureus* infection and rapamycin attenuates the phosphorylation of NF-κB p65 and STAT5 and the expression of *SLC1A3* and *SLC7A5*. The phosphorylation of mTOR, S6, NF-κB p65, and STAT5 was detected by western blot, and *SLC1A3* and *SLC7A5* were examined by RT-qPCR and western blot. **(A)** Phosphorylation of mTOR, S6, NF-κB p65, and STAT5 and levels of SLC1A3 and SLC7A5 in different BMECs treatment groups. **(B)** mRNA levels of *SLC1A3* and *SLC7A5* in different BMECs treatment groups. mTORC1 pathway was inhibited during *S. aureus* infection. The resolved bands were quantified using Gel-Pro Analyzer 4.0 (Media Cybernetics, Inc., Rockville, MD, USA). *p < 0.05; **p < 0.01. *n* = 3 independent experiments.

To verify the mTORC1 pathway regulates the expression of *SLC1A3* and *SLC7A5* through NF-κB and STAT5 is being affected during *S. aureus* infection, and demonstrate the function of mTORC1 signaling in the expression of *SLC1A3* and *SLC7A5* in BMECs, cells were managed as four groups, i.e., control cells, cells were infected by *S. aureus* 8 h, cells were treated by 100 nM rapamycin 8 h and cells were both infected by *S. aureus* and treated by 100 nM rapamycin 8 h, respectively. The phosphorylation of NF-κB and STAT5 and *SLC1A3* and *SLC7A5* expression were examined by western bolt and RT-qPCR. The results showed that the phosphorylation of mTOR, S6, NF-κB, and STAT5 was inhibited by *S. aureus* and rapamycin in BMECs (Figure 7A), and *SLC1A3* and *SLC7A5* mRNA and protein were downregulated by *S. aureus* and rapamycin (Figures 7A,B), indicating that mTORC1 pathway was inhibited during the *S. aureus* infection, and mTORC1 regulates the expression of *SLC1A3* and *SLC7A5* via NF-κB and STAT5.

**Figure 8:**
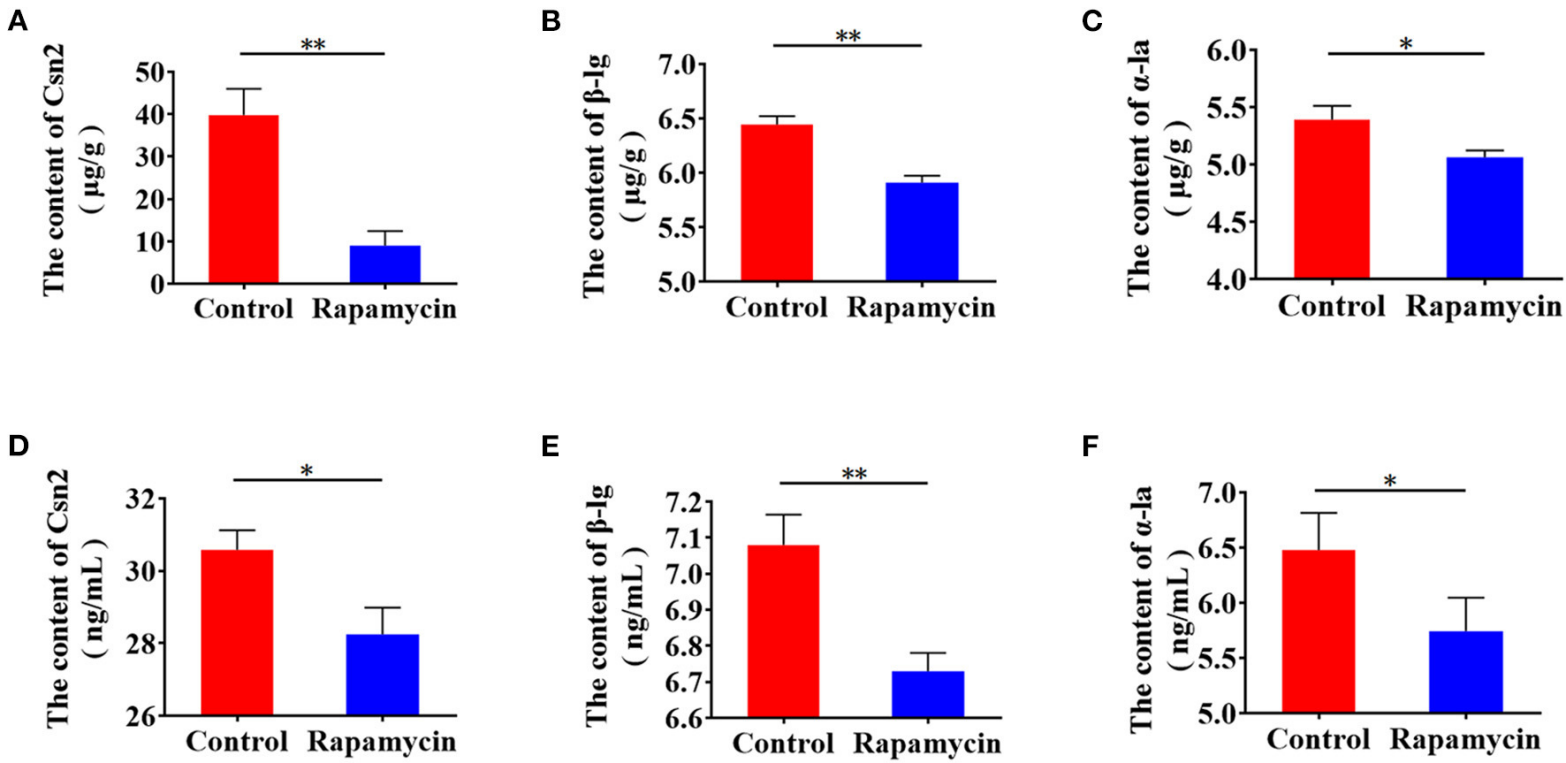
Rapamycin suppresses milk protein synthesis. **(A–C)** Intracellular Csn2 (β-casein), β-lg, and α-la after rapamycin treatment for 8 h by ELISA. **(D–F)** Csn2 (β-casein), β-lg, and α-la in cell culture medium. *p < 0.05; **p < 0.01. *n* = 3 independent experiments.

Next, to confirm the function of mTORC1 in milk protein synthesis, we measured β-casein, α-lactalbumin, and β-lactoglobulin intracellularly and in the culture medium. Milk proteins synthesis (Figures 8A–C) and secretion (Figures 8D–F) were lower, indicating that mTORC1 signaling controls milk protein synthesis in BMECs. These data demonstrate that mTORC1 governs milk protein synthesis by regulating the expression of *SLC1A3* and *SLC7A5* in BMECs.

## Discussion

*S. aureus* is the most prevalent microorganism in intramammary infections (IMIs) in dairy herds. This bacteria enters the udder and colonizes mammary tissues or invades cells, including mammary epithelial cells (54, 55). Bacteria that live in cells often cause subclinical and chronic mastitis due to their resistance to antibiotics and ability to evade phagocytosis by neutrophils (56, 57). This mastitis decreases milk production and milk quality in dairy cows (13, 14). In this study, we found that *S. aureus* causes intracellular infections in BMECs *in vitro*. *S. aureus* was internalized by BMECs over 8 h, decreasing milk protein synthesis. Further, *S. aureus* invasion affected mTORC1 signaling, and mTORC1 was activated 2 and 4 h after bacterial invasion but inhibited at 8 h. This pattern of mTORC1 activation is consistent with that of milk protein synthesis. These data indicate that the decrease in milk protein synthesis due to *S. aureus* invasion is related to mTORC1 signaling.

It is believed that a few mechanisms are involved in the decreased of milk production and milk quality in dairy cows suffer from mastitis (58), e.g., toxins, and endogenous and bacterial proteases (18, 59–61). In the present study, we found that α-hemolysin, which is the most abundant toxin in *S. aureus*, was not detectable both in cell medium and cells (Supplementary Figure S7), and the level of endogenous protease Plasmin was not increased in *S. aureus* infected cells (Supplementary Figure S2A). Meanwhile, we found that 1.5 × 10^3^ CFU/mL *S. aureus* were inoculated into medium with β-casein for 8 h, the level of β-casein did not show significant decline in infected group (Supplementary Figure S2B). These data mean that the depression of milk protein synthesis were not caused by toxins or endogenous and bacterial enzymes. Moreover, it is known that non-invasive *S. aureus* strains are also very important in *S. aureus*-mastitis. In our study, we examined the time point which *S. aureus* only adhered to BMECs but failed to internalize by referring to the Ménard's method (62). We found that *S. aureus* adhesion has no effect on the milk protein synthesis, mTORC1 signaling, the activity of NF-κB p65 and STAT5, and the expression of SLC1A3 and SLC7A5 (Supplementary Figure S6). These data demonstrated that intracellular infection of *S. aureus* caused the depression of milk protein synthesis. However, the limitation is that only one intracellular *S. aureus* strain was being consider in our work. Although the adhesion experiment was carried out in the present study, we need to reconsider non-invasive *S. aureus* strain along our work.

BMECs synthesize and secrete milk in mammary tissue and must derive exogenous amino acids from extracellular fluid to synthesize milk protein (44–46), for which various types of membrane amino acid transporters take up amino acids (47, 48). SLC (solute carrier) transporters function in many essential processes, including nutrient uptake, ion influx/efflux, and waste disposal (63). SLC1A3, also known as EAAT1 (Na^+^-dependent excitatory amino acid transporter 1) and GLAST (glutamate–aspartate transporter), has glutamate and aspartate as substrates (47). SLC7A5, also called LAT1 (L-type amino acid transporter 1), is the transport-competent unit of the LAT1/CD98 heterodimeric amino acid transporter (64) and is indispensable as a transporter of essential amino acids to maintain cell growth and protein synthesis (48). In recent years, it has been reported that amino acid transporters are related to milk protein synthesis (50, 51), but whether bacterial infection of BMECs affects the uptake of amino acid and amino acid transporter expression is unknown. In our study, we examined the expression of SLC1A3 and SLC7A5, where SLC1A3 is the transporter of Glu and Asp, and SLC7A5 is the transporter of Leu (47). Meanwhile, it is known that Glu, Asp and Leu are associated with lactation in dairy cows (65). Thus, the cells were treated with amino acids Glu, Asp and Leu, respectively, to evaluate their effects on milk protein synthesis in BMECs. Furthermore, *S. aureus* infection attenuated the expression of the amino acid transporter genes *SLC1A3* and *SLC7A5* and prevented BMECs from deriving Glu, Asp, and Leu from the culture medium, impeding amino acid induced-casein synthesis. These data indicate that *S. aureus* infection downregulates amino acid transporter genes, which are important in milk protein synthesis in BMECs.

NF-κB is a key transcription factor of inflammation-related genes and regulates the expression of EAAT1 in primary rat astrocytes and human astrocytes (66). STAT5 is critical in prolactin-induced beta-casein transcription in rodents and bovine mammary explant cultures (67), and LPS inactivates STAT5 in mouse mammary glands (68). In our study, NF-κB and STAT5 were inactivated 8 h after *S. aureus* invasion. Based these data, we conclude that *S. aureus* infection prevents amino acid uptake to suppress milk protein synthesis through impaired expression of *SLC1A3* and *SLC7A5*, which is mediated by NF-κB and STAT5 in BMECs.

## Conclusion

*S. aureus* can be internalized by BMECs *in vitro*, and the internalized bacteria can undergo intracellular proliferation. Milk proteins were suppressed 8 h after *S. aureus* invasion. *S. aureus* invasion downregulated the amino acid transporter genes *SLC1A3* and *SLC7A5*, impaired absorption of amino acids by BMECs from the culture medium, decreased exogenous amino acid-induced β-casein synthesis, and attenuated mTORC1 signaling. Rapamycin inhibited the activation of NF-κB and STAT5, the expression of *SLC1A3* and *SLC7A5*, and milk protein synthesis. The mechanism by which *S. aureus* infection depresses milk protein synthesis in BMECs is likely *S. aureus* invasion-mediated attenuation of mTORC1 signaling and SLC1A3 and SLC7A5 expression, resulting in suppression of amino acid uptake and milk protein synthesis.

## Data Availability Statement

The original contributions presented in the study are included in the article/Supplementary Material, further inquiries can be directed to the corresponding author/s.

## Ethics Statement

The animal study was reviewed and approved by Inner Mongolia University Animal Care and Use Committee.

## Author Contributions

ZW and YW proposed the initial experiments and analyzed the experimental feasibility. YC, YM, and XF performed the experiments. TL participated in the design of experimental technical route. XY cultured the primary BMECs. QJ, RY, and XC analyzed the experimental data together. YC wrote the final manuscript. YW revised the final manuscript. All authors approved the final article.

## Funding

This work was supported by the Natural Sciences Foundation of China (No. 31960669), the Natural Sciences Foundation of Inner Mongolia (Nos. 2020MS03021 and 2021MS08065), the Scientific Research Projects in Higher Education Institutions of Inner Mongolia (No. NJZY19235), and the Science and Technology Major Project of Inner Mongolia Autonomous Region of China to the State Key Laboratory of Reproductive Regulation and Breeding of Grassland Livestock (No. zdzx2018065).

## Conflict of Interest

The authors declare that the research was conducted in the absence of any commercial or financial relationships that could be construed as a potential conflict of interest.

## Publisher's Note

All claims expressed in this article are solely those of the authors and do not necessarily represent those of their affiliated organizations, or those of the publisher, the editors and the reviewers. Any product that may be evaluated in this article, or claim that may be made by its manufacturer, is not guaranteed or endorsed by the publisher.

## Supplementary Material

Supplementary Figure S1 *Staphylococcus aureus* invades BMECs. BMECs were infected with *S. aureus* for 2 h, and the cells were cultured in medium supplemented with antibiotics and lysozyme to kill and lyse the extracellular bacteria. **(A)** Intracellular *S. aureus* (green) stained with CFSE by laser confocal microscopy; BMEC nuclei were co-stained with DAPI (blue), and actin was stained with phalloidin (red). Scale bars represent 20 μm. **(B)** *S. aureus* was internalized by BMECs, based on micrographs obtained by TEM; several important observations are magnified. Red arrows indicate *S. aureus*. a Control, uninfected BMECs, b *S. aureus* in cytosolic vacuoles by TEM in BMECs. Scale bars represent 2 μm. *N* = 3 independent experiments.

Supplementary Figure S2 *Staphylococcus aureus* adhesion does not affect the contents of protease Plasmin in BMECs 8 h after infection, and β-casein was not degraded by *S. aureus*. **(A)** Levels of Plasmin (Pla) in intracellular and cell culture medium. **(B)** Levels of Csn2 (β-casein) in co-incubated culture medium. ns *p* > 0.05. *n* = 3 independent experiments.

Supplementary Figure S3 Cell apoptosis was not found in BMECs after *Staphylococcus aureus* infection 8 h. **(A)** Hoechst assay was used to examine apoptosis in infected BMECs and control. **(B)** FITC annexin V apoptosis detection was used to examine apoptosis in infected BMECs and control. **(C)** The expression levels of Caspase 3 and cleaved Caspase 3 in BMECs were examined by Western blotting. The resolved bands were quantified using Gel-Pro Analyzer 4.0 (Media Cybernetics, Inc., Rockville, MD, USA). ns *p* > 0.05. *n* = 3 independent experiments.

Supplementary Figure S4 Putative transcription factor binding sites (TFBSs) for NF-κB and STAT5 in the promoters of *SLC1A3* and *SLC7A5*. NF-κB TFBS in the promoter sequences of **(A)** *SLC1A3* and **(B)** *SLC7A5*. STAT5a TFBS in the promoter sequences of **(C)** *SLC1A3* and **(D)** *SLC7A5*.

Supplementary Figure S5 Putative transcription factor binding motifs (TFBMs) for NF-κB and STAT5 in the promoters of *SLC1A3* and *SLC7A5* (red boxes represent motif). NF-κB TFBM in the promoter sequence of **(A)** *SLC1A3* (−1878, −1848, −1797, −1780,−1749, −1276, −524, −102, −80, 79, 80, *p* < 0.001) and **(B)** *SLC7A5* (−159,−211,−396, −507,−605, −692, −737, −782, −1073, −1284, −1322, −1371,−1573, *p* < 0.001). STAT5a TFBM in the promoter sequence of **(C)** *SLC1A3* (-1432, −1254,−1005,−844,−442, −354, −114, 75, *p* < 0.001) and **(D)** *SLC7A5* (-1018,−1067,−1206,−1768, *p* < 0.001). TFBM of **(E)** NF-κB and **(F)** STAT5a in the Jaspar Database.

Supplementary Figure S6 *Staphylococcus aureus* adhesion has no effect on the milk protein synthesis, mTORC1 signaling, the activity of NF-κB p65 and STAT5, and the expression of SLC1A3 and SLC7A5. **(A)** Levels of Csn2 (β-casein), β-lg and α-la in cell culture medium. **(B,C)** Phosphorylation of mTOR, S6, NF-κB p65, and STAT5 and the expression of SLC1A3 and SLC7A5. ns *p* > 0.05. *n* = 3 independent experiments.

Supplementary Figure S7 *Staphylococcus aureus* toxin alpha-hemolysin (Hlα) was determined by ELISA after infection 8 h. The α-hemolysin was not detectable both in cell medium and cells. ns *p* > 0.05. *n* = 3 independent experiments.

Supplementary Table S1 The target genes and primers for qPCR.

Supplementary Table S2 The colon number of *S. aureus* in BMECs and cells medium ($\bar{\text{x}}$ ± SD).

## Abbreviations

mTOR: mechanistic target of rapamycin
mTORC1: mTOR complex 1
S6K1: ribosomal protein S6 kinase 1
4E-BP1: eukaryotic translation initiation factor 4E-binding protein 1
BMECs: bovine mammary epithelial cells
CM: clinical mastitis
SM: subclinical mastitis
NF-κB: nuclear factor kappa-B
STAT: signal transducer and activator of transcription.

## References

[1] Ramírez-Rivera EJ Rodríguez-Miranda J Huerta-Mora IR Cárdenas-Cágal A Juárez-Barrientos JM. Tropical milk production systems and milk quality: a review. Trop Anim Health Prod. (2019) 51:1295–305. 10.1007/s11250-019-01922-1 31134554

[2] Singhal S Baker RD Baker SS. A comparison of the nutritional value of cow's milk and nondairy beverages. J Pediatr Gastroenterol Nutr. (2017) 64:799–805. 10.1097/MPG.0000000000001380 27540708

[3] Farrell HM Jr Jimenez-Flores R Bleck GT Brown EM Butler JE Creamer LK. Nomenclature of the proteins of cows' milk–sixth revision. J Dairy Sci. (2004) 87:1641–74. 10.3168/jds.S0022-0302(04)73319-6 15453478

[4] Maurmayr A Pegolo S Malchiodi F Bittante G Cecchinato A. Milk protein composition in purebred Holsteins and in first/second-generation crossbred cows from Swedish Red, Montbeliarde and Brown Swiss bulls. Animal. (2018) 12:2214–20. 10.1017/S1751731117003640 29307328

[5] Bonfatti V Grigoletto L Cecchinato A Gallo L Carnier P. Validation of a new reversed-phase high-performance liquid chromatography method for separation and quantification of bovine milk protein genetic variants. J Chromatogr A. (2008) 1195:101–6. 10.1016/j.chroma.2008.04.075 18495141

[6] Brinkmann J Jagannathan V Drögemüller C Rieder S Leeb T Thaller G. Genetic variability of the equine casein genes. J Dairy Sci. (2016) 99:5486–97. 10.3168/jds.2015-10652 27108172

[7] Holt C Carver JA Ecroyd H Thorn DC. Invited review: Caseins and the casein micelle: their biological functions, structures, and behavior in foods. J Dairy Sci. (2013) 96:6127–46. 10.3168/jds.2013-6831 23958008

[8] Whist AC Osterås O Sølverød L. Association between isolation of *Staphylococcus aureus* one week after calving and milk yield, somatic cell count, clinical mastitis, and culling through the remaining lactation. J Dairy Res. (2009) 76:24–35. 10.1017/S0022029908003592 18922193

[9] Heikkilä AM Liski E Pyörälä S Taponen S. Pathogen-specific production losses in bovine mastitis. J Dairy Sci. (2018) 101:9493–504. 10.3168/jds.2018-14824 30122416

[10] Botaro BG Cortinhas CS Dibbern AG. e Silva LF, Benites NR, Dos Santos MV. Staphylococcus aureus intramammary infection affects milk yield and SCC of dairy cows. Trop Anim Health Prod. (2015) 47:61–6. 10.1007/s11250-014-0683-5 25319448

[11] Tesfaye GY Regassa FG Kelay B. Milk yield and associated economic losses in quarters with subclinical mastitis due to *Staphylococcus aureus* in Ethiopian crossbred dairy cows. Trop Anim Health Prod. (2010) 42:925–31. 10.1007/s11250-009-9509-2 20012690

[12] Gröhn YT Wilson DJ González RN Hertl JA Schulte H Bennett G. Effect of pathogen-specific clinical mastitis on milk yield in dairy cows. J Dairy Sci. (2004) 87:3358–74. 10.3168/jds.S0022-0302(04)73472-4 15377615

[13] Nagasawa Y Kiku Y Sugawara K Tanabe F Hayashi T. Exfoliation rate of mammary epithelial cells in milk on bovine mastitis caused by *Staphylococcus aureus* is associated with bacterial load. Anim Sci J. (2018) 89:259–66. 10.1111/asj.12886 28891152

[14] Reksen O Sølverød L Østerås O. Relationships between milk culture results and milk yield in Norwegian dairy cattle. J Dairy Sci. (2007) 90:4670–8. 10.3168/jds.2006-900 17881688

[15] Kayano M Itoh M Kusaba N Hayashiguchi O Kida K Tanaka Y. Associations of the first occurrence of pathogen-specific clinical mastitis with milk yield and milk composition in dairy cows. J Dairy Res. (2018) 85:309–16. 10.1017/S0022029918000456 30101726

[16] Mansor R Mullen W Albalat A Zerefos P Mischak H Barrett DC. A peptidomic approach to biomarker discovery for bovine mastitis. J Proteomics. (2013) 85:89–98. 10.1016/j.jprot.2013.04.027 23639846

[17] Hogarth CJ Fitzpatrick JL Nolan AM Young FJ Pitt A Eckersall PD. Differential protein composition of bovine whey: a comparison of whey from healthy animals and from those with clinical mastitis. Proteomics. (2004) 4:2094–100. 10.1002/pmic.200300723 15221770

[18] Johansson M Åkerstedt M Li S Zamaratskaia G Sternesjö Lundh Å. Casein breakdown in bovine milk by a field strain of *Staphylococcus aureus*. J Food Prot. (2013) 76:1638–42. 10.4315/0362-028X.JFP-13-112 23992512

[19] Åkerstedt M Wredle E Lam V Johansson M. Protein degradation in bovine milk caused by *Streptococcus agalactiae*. J Dairy Res. (2012) 79:297–303. 10.1017/S0022029912000301 22850579

[20] Kitchen BJ. Review of the progress of dairy science: bovine mastitis: milk compositional changes and related diagnostic tests. J Dairy Res. (1981) 48:167–88. 10.1017/S0022029900021580 7021617

[21] Paludetti LF Kelly AL O'Brien B Jordan K Gleeson D. Microbiological quality of milk from farms to milk powder manufacture: an industrial case study. J Dairy Res. (2019) 86:242–7. 10.1017/S0022029919000347 31156075

[22] Aryana KJ Olson DW. A 100-Year Review: Yogurt and other cultured dairy products. J Dairy Sci. (2017) 100:9987–10013. 10.3168/jds.2017-12981 29153184

[23] Miglior F Fleming A Malchiodi F Brito LF Martin P Baes CF. 100-Year Review: Identification and genetic selection of economically important traits in dairy cattle. J Dairy Sci. (2017) 100:10251–71. 10.3168/jds.2017-12968 29153164

[24] Chen J Ou Y Yang Y Li W Xu Y Xie Y. KLHL22 activates amino-acid-dependent mTORC1 signaling to promote tumorigenesis and ageing. Nature. (2018) 557:585–9. 10.1038/s41586-018-0128-9 29769719

[25] Saxton RA Sabatini DM. mTOR signaling in growth, metabolism, and disease. Cell. (2017) 169:361–71. 10.1016/j.cell.2017.03.035 28388417

[26] Mutvei AP Nagiec MJ Hamann JC Kim SG Vincent CT Blenis J. Rap1-GTPases control mTORC1 activity by coordinating lysosome organization with amino acid availability. Nat Commun. (2020) 11:1416. 10.1038/s41467-020-15156-5 32184389 PMC7078236

[27] Rabanal-Ruiz Y Otten EG1 Korolchuk VI. mTORC1 as the main gateway to autophagy. Essays Biochem. (2017) 61:565–84. 10.1042/EBC20170027 29233869 PMC5869864

[28] Demetriades C Doumpas N Teleman AA. Regulation of TORC1 in response to amino acid starvation via lysosomal recruitment of TSC2. Cell. (2014) 156:786–99. 10.1016/j.cell.2014.01.024 24529380 PMC4346203

[29] Menon S Dibble CC Talbott G Hoxhaj G Valvezan AJ Takahashi H. Spatial control of the TSC complex integrates insulin and nutrient regulation of mTORC1 at the lysosome. Cell. (2014) 156:771–85. 10.1016/j.cell.2013.11.049 24529379 PMC4030681

[30] Kim E Goraksha-Hicks P Li L Neufeld TP Guan KL. Regulation of TORC1 by Rag GTPases in nutrient response. Nat Cell Biol. (2008) 10:935–45. 10.1038/ncb1753 18604198 PMC2711503

[31] Sancak Y Peterson TR Shaul YD Lindquist RA Thoreen CC Bar-Peled L. The Rag GTPases bind raptor and mediate amino acid signaling to mTORC1. Science. (2008) 320:1496–501. 10.1126/science.1157535 18497260 PMC2475333

[32] Gu X Orozco JM Saxton RA Condon KJ Liu GY Krawczyk PA. SAMTOR is an S-adenosylmethionine sensor for the mTORC1 pathway. Science. (2017) 358:813–8. 10.1126/science.aao3265 29123071 PMC5747364

[33] Saxton RA Chantranupong L Knockenhauer KE Schwartz TU Sabatini DM. Mechanism of arginine sensing by CASTOR1 upstream of mTORC1. Nature. (2016) 536:229–33. 10.1038/nature19079 27487210 PMC4988899

[34] Jewell JL Kim YC Russell RC Yu FX Park HW Plouffe SW. Differential regulation of mTORC1 by leucine and glutamine. Science. (2015) 347:194–8. 10.1126/science.1259472 25567907 PMC4384888

[35] Rebsamen M Pochini L Stasyk T de Araújo ME Galluccio M Kandasamy RK. SLC38A9 is a component of the lysosomal amino acid sensing machinery that controls mTORC1. Nature. (2015) 519:477–81. 10.1038/nature14107 25561175 PMC4376665

[36] Zhang Y Nicholatos J Dreier JR Ricoult SJ Widenmaier SB Hotamisligil GS. Coordinated regulation of protein synthesis and degradation by mTORC1. Nature. (2014) 513:440–3. 10.1038/nature13492 25043031 PMC4402229

[37] Hu L Chen Y Cortes IM Coleman DN Dai H Liang Y. Supply of methionine and arginine alters phosphorylation of mechanistic target of rapamycin (mTOR), circadian clock proteins, and α-s1-casein abundance in bovine mammary epithelial cells. Food Funct. (2020) 11:883–94. 10.1039/C9FO02379H 31942894

[38] Zhang MC Zhao SG Wang SS Luo CC Gao HN Zheng N. D-Glucose and amino acid deficiency inhibits casein synthesis through JAK2/STAT5 and AMPK/mTOR signaling pathways in mammary epithelial cells of dairy cows. J Dairy Sci. (2018) 101:1737–46. 10.3168/jds.2017-12926 29248227

[39] Qi H Meng C Jin X Li X Li P Gao X. Methionine promotes milk protein and fat synthesis and cell proliferation via the SNAT2-PI3K signaling pathway in bovine mammary epithelial cells. J Agric Food Chem. (2018) 66:11027–33. 10.1021/acs.jafc.8b04241 30274521

[40] Dai W Zhao F Liu J Liu H. ASCT2 is involved in SARS-mediated β-casein synthesis of bovine mammary epithelial cells with methionine supply. J Agric Food Chem. (2020) 68:13038–45. 10.1021/acs.jafc.9b03833 31597423

[41] Liu Y Hou J Zhang M Seleh-Zo E Wang J Cao B. circ-016910 sponges miR-574-5p to regulate cell physiology and milk synthesis via MAPK and PI3K/AKT-mTOR pathways in GMECs. J Cell Physiol. (2020) 235:4198–216. 10.1002/jcp.29370 31663119 PMC7028128

[42] Zhao Y Yan S Chen L Shi B Guo X. Effect of interaction between leucine and acetate on the milk protein synthesis in bovine mammary epithelial cells. Anim Sci J. (2019) 90:81–9. 10.1111/asj.13125 30397989

[43] Sigl T Meyer HH Wiedemann S. Gene expression analysis of protein synthesis pathways in bovine mammary epithelial cells purified from milk during lactation and short-term restricted feeding. J Anim Physiol Anim Nutr (Berl). (2014) 98:84–95. 10.1111/jpn.12039 23402545

[44] Luo C Zheng N Zhao S Wang J. Sestrin2 negatively regulates casein synthesis through the SH3BP4-mTORC1 pathway in response to aa depletion or supplementation in cow mammary epithelial cells. J Agric Food Chem. (2019) 67:4849–59. 10.1021/acs.jafc.9b00716 30969118

[45] Cant JP Kim JJM Cieslar SRL Doelman J. Symposium review: Amino acid uptake by the mammary glands: Where does the control lie? J Dairy Sci. (2018) 101:5655–66. 10.3168/jds.2017-13844 29605320

[46] Bionaz M Loor JJ. Gene networks driving bovine mammary protein synthesis during the lactation cycle. Bioinform Biol Insights. (2011) 5:83–98. 10.4137/BBI.S7003 21698073 PMC3118679

[47] Yahyaoui R Pérez-Frías J. Amino acid transport defects in human inherited metabolic disorders. Int J Mol Sci. (2019) 21:E119. 10.3390/ijms21010119 31878022 PMC6981491

[48] Lin Y Duan X Lv H Yang Y Liu Y Gao X. The effects of L-type amino acid transporter 1 on milk protein synthesis in mammary glands of dairy cows. J Dairy Sci. (2018) 101:1687–96. 10.3168/jds.2017-13201 29224866

[49] Dai W Zhao F Liu J Liu H. Seryl-tRNA synthetase is involved in methionine stimulation of β-casein synthesis in bovine mammary epithelial cells. Br J Nutr. (2020) 123:489–98. 10.1017/S0007114519002885 31711551 PMC7015878

[50] Duan X Lin Y Lv H Yang Y Jiao H Hou X. Methionine induces LAT1 expression in dairy cow mammary gland by activating the mTORC1 signaling pathway. DNA Cell Biol. (2017) 36:1126–33. 10.1089/dna.2017.3792 29040000

[51] Saad A Bijian K Qiu D da Silva SD Marques M Chang CH. Insights into a novel nuclear function for Fascin in the regulation of the amino-acid transporter SLC3A2. Sci Rep. (2016) 6:36699. 10.1038/srep36699 27819326 PMC5098188

[52] Guo Z Zhao K Feng X Yan D Yao R Chen Y. mTORC2 regulates lipogenic gene expression through PPAR?? to control lipid synthesis in bovine mammary epithelial cells. BioMed Research International. (2019) 5196028. 10.1155/2019/5196028 31223619 PMC6541957

[53] Guo Z Cheng X Feng X Zhao K Zhang M Yao R. The mTORC1/4EBP1/PPARγ axis mediates insulin-induced lipogenesis by regulating lipogenic gene expression in bovine mammary epithelial cells. J Agric Food Chem. (2019) 67:6007–18. 10.1021/acs.jafc.9b01411 31060359

[54] Piccinini R Tassi R Daprà V Pilla R Fenner J Carter B. Study of *Staphylococcus aureus* collected at slaughter from dairy cows with chronic mastitis. J Dairy Res. (2012) 79:249–55. 10.1017/S002202991200009X 22369758

[55] Bohl LP Isaac P Breser ML Orellano MS Correa SG Tolosa de Talamoni NG. Interaction between bovine mammary epithelial cells and planktonic or biofilm Staphylococcus aureus: The bacterial lifestyle determines its internalization ability and the pathogen recognition. Microb Pathog. (2021) 152:104604. 10.1016/j.micpath.2020.104604 33186743

[56] Rajeeve K Das S Prusty BK Rudel T. Chlamydia trachomatis paralyses neutrophils to evade the host innate immune response. Nat Microbiol. (2018) 3:824–35. 10.1038/s41564-018-0182-y 29946164

[57] Watkins KE Unnikrishnan M. Evasion of host defenses by intracellular *Staphylococcus aureus*. Adv Appl Microbiol. (2020) 112:105–41. 10.1016/bs.aambs.2020.05.001 32762866

[58] Martins L Barcelos MM Cue RI Anderson KL Santos MVD Goncalves JL. Chronic subclinical mastitis reduces milk and components yield at the cow level. J Dairy Res. (2020) 87:98–305. 10.1017/S0022029920000321 32398175

[59] Lahteenmaki K Kuusela P Korhonen TK. Bacterial plasminogen activators and receptors. FEMS Microbiol Rev. (2001) 25:531–52. 10.1016/S0168-6445(01)00067-5 11742690

[60] Kelly AL O'Flaherty F Fox PF. Indigenous proteolytic enzymes in milk: A brief overview of the present state of knowledge. Int Dairy J. (2006) 16:563–72. 10.1016/j.idairyj.2005.10.019

[61] Livney YD Schwan AL Dalgleish DG. A study of beta-casein tertiary structure by intramolecular crosslinking and mass spectrometry. J Dairy Sci. (2004) 87:3638–47. 10.3168/jds.S0022-0302(04)73502-X 15483147

[62] Ménard G Bonnaure-Mallet M Donnio PY. Adhesion of Staphylococcus aureus to epithelial cells: an *in vitro* approach to study interactions within the nasal microbiota. J Med Microbiol. (2020) 69:1253–61. 10.1099/jmm.0.001248 32909934

[63] Song W Li D Tao L Luo Q Chen L. Solute carrier transporters: the metabolic gatekeepers of immune cells. Acta Pharm Sin B. (2020) 10:61–78. 10.1016/j.apsb.2019.12.006 31993307 PMC6977534

[64] Napolitano L Scalise M Galluccio M Pochini L Albanese LM Indiveri C. LAT1 is the transport competent unit of the LAT1/CD98 heterodimeric amino acid transporter. Int J Biochem Cell Biol. (2015) 67:25–33. 10.1016/j.biocel.2015.08.004 26256001

[65] Mackle TR Dwyer DA Ingvartsen KL Chouinard PY Ross DA Bauman DE. Evaluation of whole blood and plasma in the interorgan supply of free amino acids for the mammary gland of lactating dairy cows. J Dairy Sci. (2000) 83:1300–09. 10.3168/jds.S0022-0302(00)74996-4 10877395

[66] Karki P Kim C Smith K Son DS Aschner M Lee E. Transcriptional regulation of the astrocytic excitatory amino acid transporter 1 (EAAT1) via NF-kappa B and Yin Yang 1 (YY1). J Biol Chem. (2015) 290:23725–37. 10.1074/jbc.M115.649327 26269591 PMC4583050

[67] Yang J Kennelly JJ Baracos VE. The activity of transcription factor Stat5 responds to prolactin, growth hormone, and IGF-I in rat and bovine mammary explant culture. J Anim Sci. (2000) 78:3114–25. 10.2527/2000.78123114x 11132826

[68] Kobayashi K Oyama S Uejyo T Kuki C Rahman MM Kumura H. Underlying mechanisms involved in the decrease of milk secretion during *Escherichia coli* endotoxin induced mastitis in lactating mice. Vet Res. (2013) 44:119. 10.1186/1297-9716-44-119 24308795 PMC4028753

