# Supplementary figures and images for: Intracellular Staphylococcus aureus Infection Decreases Milk Protein Synthesis by Preventing Amino Acid Uptake in Bovine Mammary Epithelial Cells
Source: Front Vet Sci. 2021 Nov 16;8:756375. doi: 10.3389/fvets.2021.756375 (PMC8636274; doi:10.3389/fvets.2021.756375)

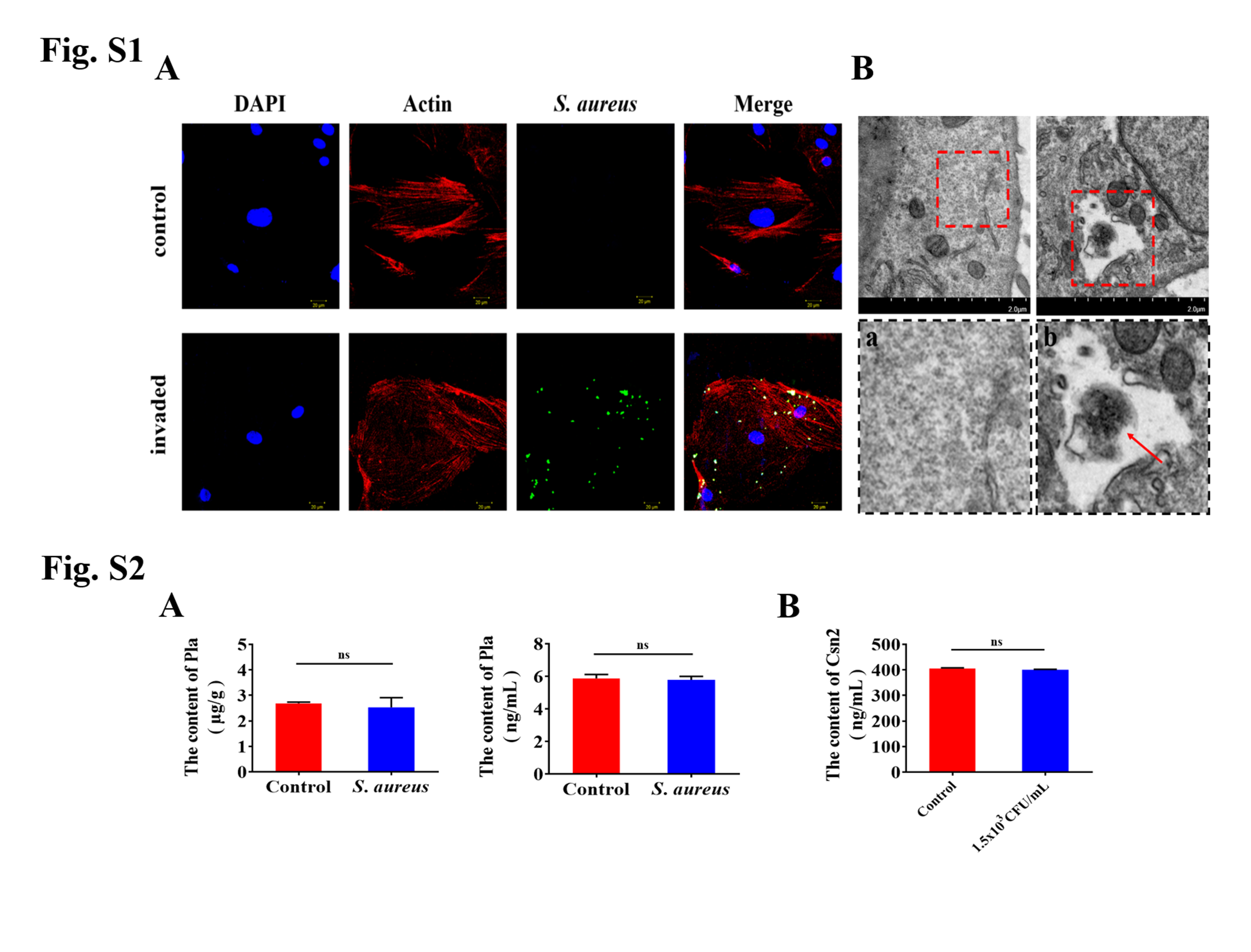

Supplement: Supplementary Figure S1 — Staphylococcus aureus invades BMECs. BMECs were infected with S. aureus for 2 h, and the cells were cultured in medium supplemented with antibiotics and lysozyme to kill and lyse the extracellular bacteria. (A) Intracellular S. aureus (green) stained with CFSE by laser confocal microscopy; BMEC nuclei were co-stained with DAPI (blue), and actin was stained with phalloidin (red). Scale bars represent 20 μm. (B) S. aureus was internalized by BMECs, based on micrographs obtained by TEM; several important observations are magnified. Red arrows indicate S. aureus. a Control, uninfected BMECs, b S. aureus in cytosolic vacuoles by TEM in BMECs. Scale bars represent 2 μm. N = 3 independent experiments. [file Image_1.tif]

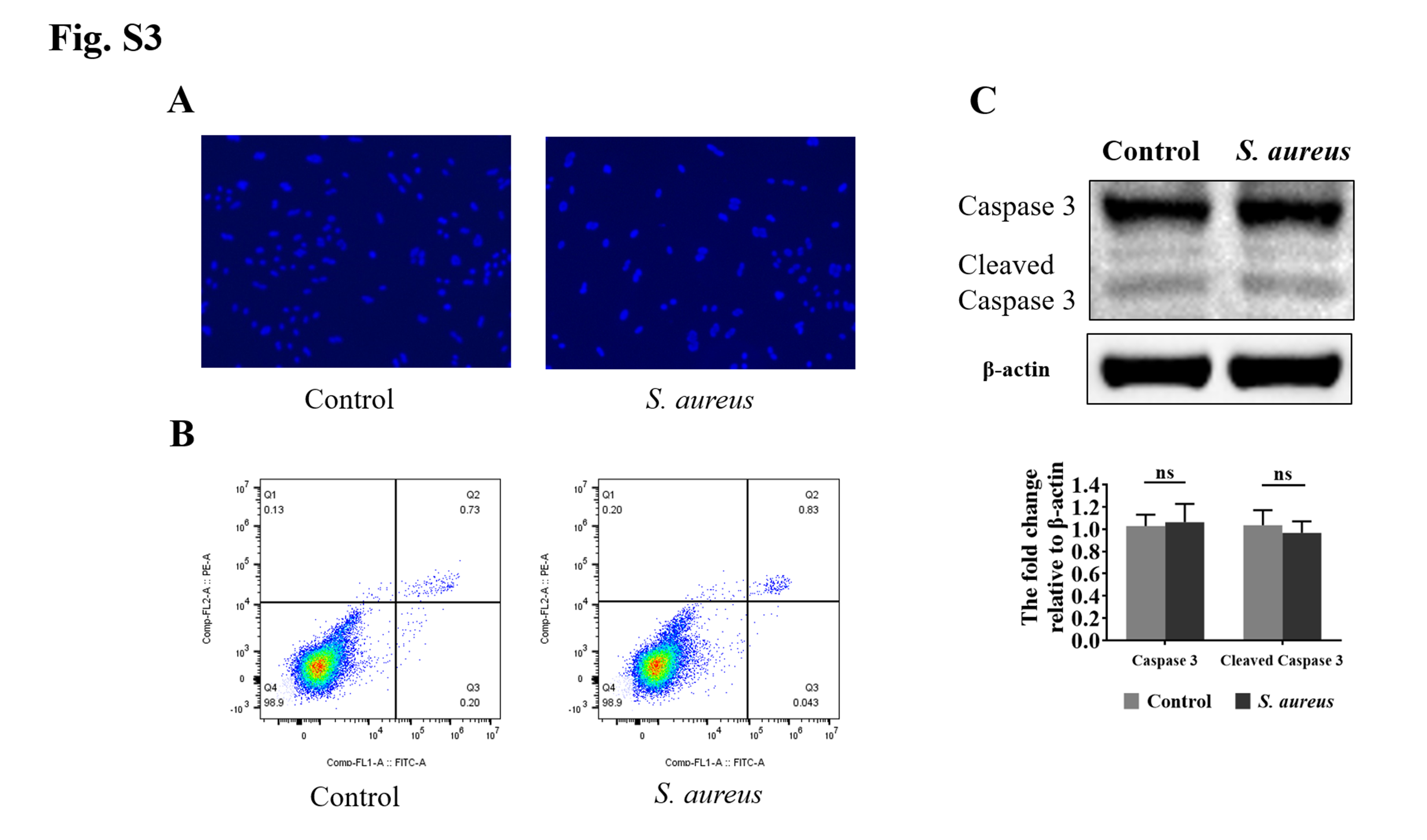

Supplement: Supplementary Figure S3 — Cell apoptosis was not found in BMECs after Staphylococcus aureus infection 8 h. (A) Hoechst assay was used to examine apoptosis in infected BMECs and control. (B) FITC annexin V apoptosis detection was used to examine apoptosis in infected BMECs and control. (C) The expression levels of Caspase 3 and cleaved Caspase 3 in BMECs were examined by Western blotting. The resolved bands were quantified using Gel-Pro Analyzer 4.0 (Media Cybernetics, Inc., Rockville, MD, USA). ns p > 0.05. n = 3 independent experiments. [file Image_3.tif]

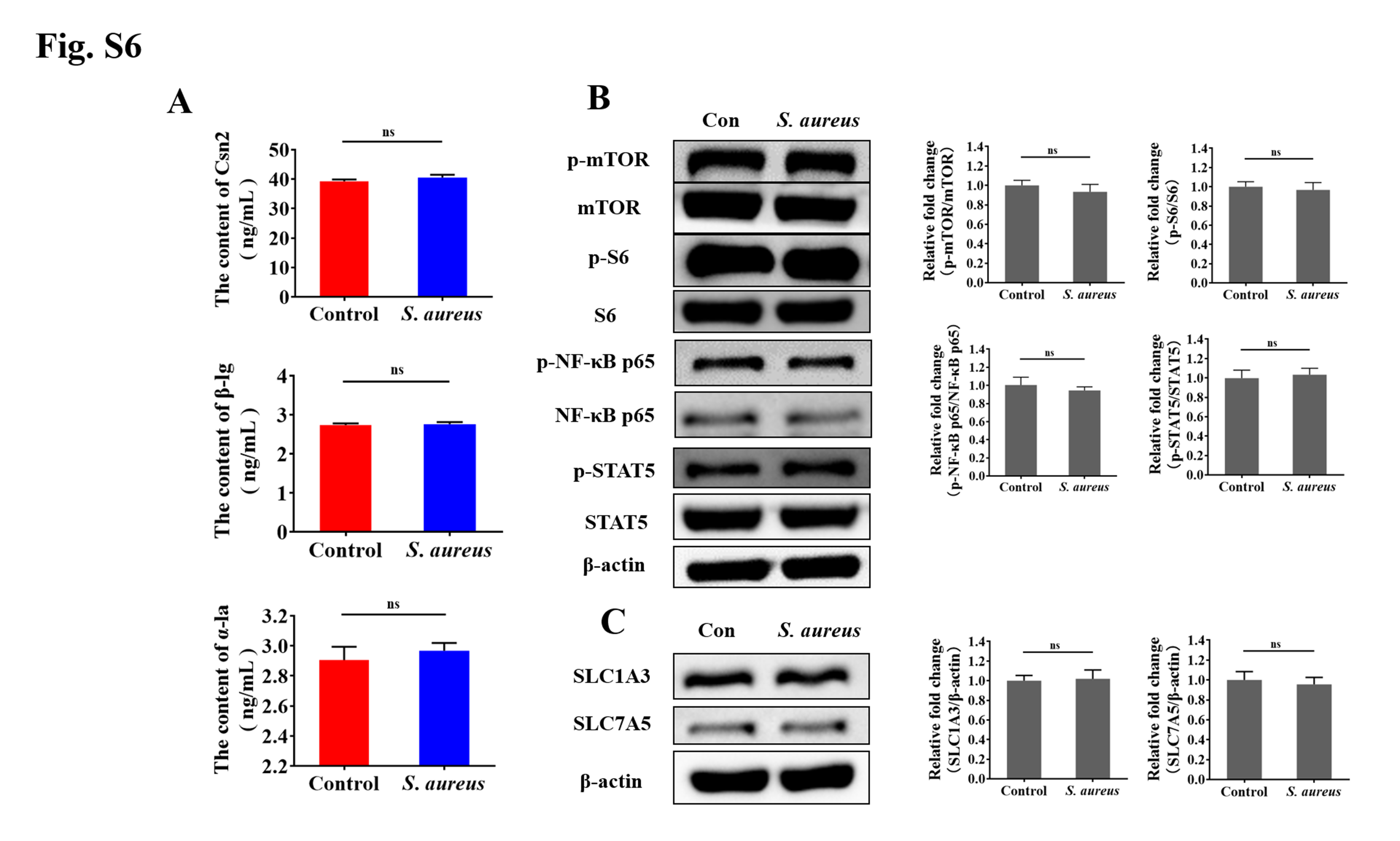

Supplement: Supplementary Figure S6 — Staphylococcus aureus adhesion has no effect on the milk protein synthesis, mTORC1 signaling, the activity of NF-κB p65 and STAT5, and the expression of SLC1A3 and SLC7A5. (A) Levels of Csn2 (β-casein), β-lg and α-la in cell culture medium. (B,C) Phosphorylation of mTOR, S6, NF-κB p65, and STAT5 and the expression of SLC1A3 and SLC7A5. ns p > 0.05. n = 3 independent experiments. [file Image_6.tif]

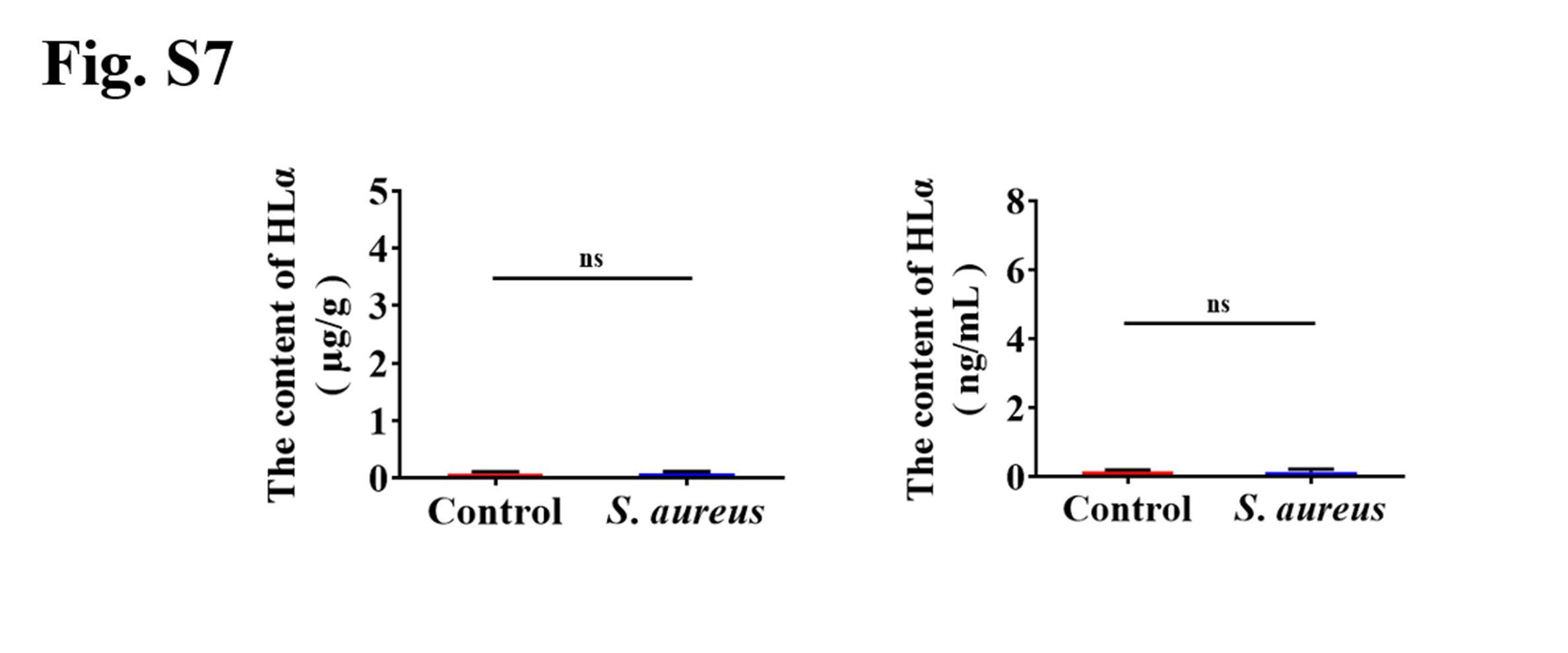

Supplement: Supplementary Figure S7 — Staphylococcus aureus toxin alpha-hemolysin (Hlα) was determined by ELISA after infection 8 h. The α-hemolysin was not detectable both in cell medium and cells. ns p > 0.05. n = 3 independent experiments. [file Image_7.tif]
