# Supplementary material for: Intracellular Staphylococcus aureus Infection Decreases Milk Protein Synthesis by Preventing Amino Acid Uptake in Bovine Mammary Epithelial Cells
Source: Front Vet Sci. 2021 Nov 16;8:756375. doi: 10.3389/fvets.2021.756375 (PMC8636274; doi:10.3389/fvets.2021.756375)
Supplement: Supplementary Figure S4 — Putative transcription factor binding sites (TFBSs) for NF-κB and STAT5 in the promoters of SLC1A3 and SLC7A5. NF-κB TFBS in the promoter sequences of (A) SLC1A3 and (B) SLC7A5. STAT5a TFBS in the promoter sequences of (C) SLC1A3 and (D) SLC7A5. [file Data_Sheet_1.pdf]

Supplementary File 1

Prediction of putative transcription factor binding sites (TFBS) and the transcription factor binding motifs (TFBM) of the transcription factor NF-κB and STAT5 in the promoter sequence of the *SLC1A3* and *SLC7A5*.

The TFBS of NF-κB and STAT5 was analyzed in the promoter sequence of the *SLC1A3* and *SLC7A5* by bioinformatics analysis using the UCSC Genome Browser (http://genome.ucsc.edu/index.html) and the PROMO database (http://alggen.lsi.upc.es/cgi-bin/promo_v3/promo/promoinit.cgi?dirDB=TF_8.3), and as a result, the TFBS of the NF-κB was found in the promoter sequence of the *SLC1A3* (Figure S2(A)) and *SLC7A5* (Figure S2(B)), respectively. The TFBS of the STAT5 was found in the promoter sequence of the *SLC1A3* (Figure S2(C)) and *SLC7A5* (Figure S2(D)), respectively. The UCSC Genome Browser was used to find the promoter sequences of the *SLC1A3* and *SLC7A5*, and the PROMO database was used to identify the putative transcription factor binding sites (TFBS) of NF-κB and STAT5 in the promoter sequence of *SLC1A3* and *SLC7A5*, respectively. The transcription factor NF-κB and STAT5 was predicted within a dissimilarity margin less or equal than 15%.

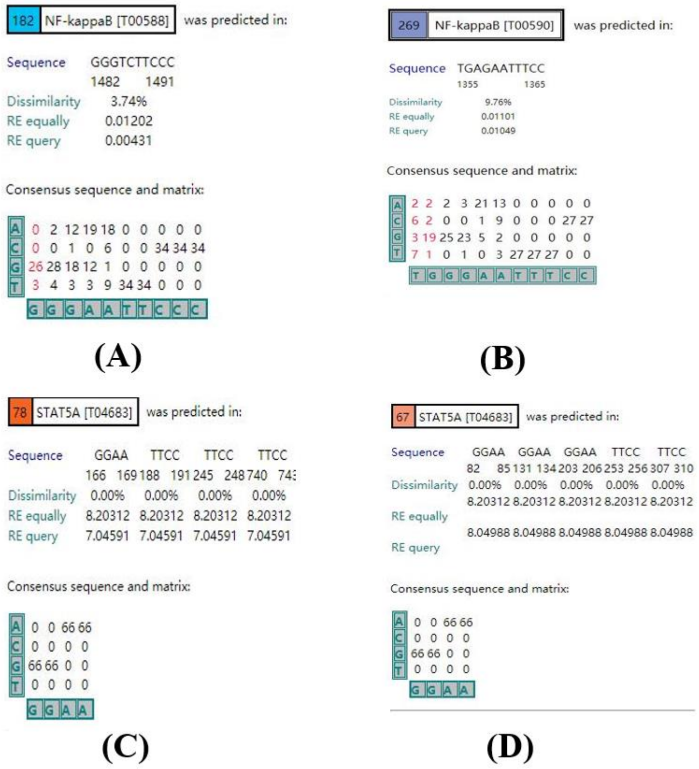

FIGURE S4 The putative transcription factor binding sites (TFBS) of NF-κB and STAT5 in the promoter sequence of *SLC1A3* and *SLC7A5*.

(A) The NF-κB TFBS in the promoter sequence of *SLC1A3*.

(B) The NF-κB TFBS in the promoter sequence of *SLC7A5*.

(C) The STAT5a TFBS in the promoter sequence of *SLC1A3*.

(D) The STAT5a TFBS in the promoter sequence of *SLC7A5*.

The TFBM of NF-κB and STAT5 were predicted in the promoter sequence of the *SLC1A3* and *SLC7A5* by bioinformatics analysis using the Eukaryotic Promoter Database (https://epd.epfl.ch//index.php) and the Jaspar Database (http://jaspar.genereg.net/). The promoter sequences were selected from -2000 to 100 bp relative to TSS and a cut-off, using Eukaryotic Promoter Database. The TFBM of the NF-κB and STAT5 were defined by Jaspar Database, respectively. And as a consequence, the motifs of the NF-κB was found in the promoter sequence of the *SLC1A3* (Figure S3(A)), *SLC7A5* (Figure S3(B)), and the motifs of the STAT5 was found in the promoter sequence of the *SLC1A3* (Figure S3(C)) and *SLC7A5* (Figure S3(D)) (p < 0.001). The TFBM of NF-κB of Jaspar Database (E) and the TFBM of STAT5a of Jaspar Database (F) were exhibited.

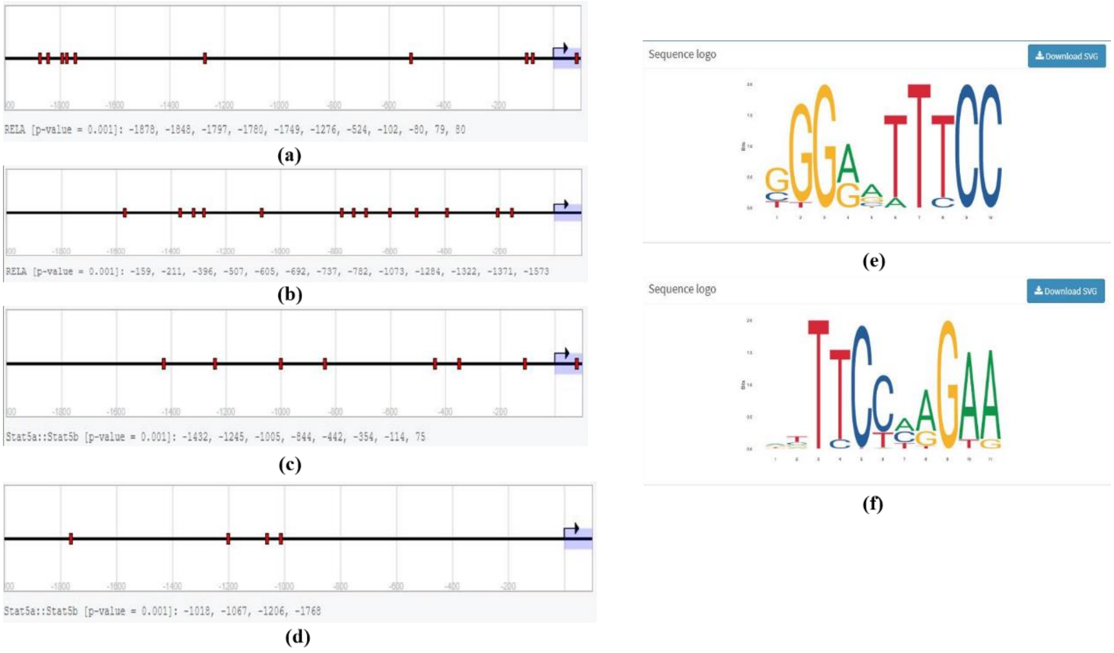

FIGURE S5 The putative transcription factor binding motifs (TFBM) of NF-κB and STAT5 in the promoter sequence of *SLC1A3* and *SLC7A5* (Red boxes represent motif). (A) The NF-κB TFBM in the promoter sequence of *SLC1A3* (-1878, -1848, -1797, -1780, -1749, -1276, -524, -102, -80, 79, 80, p < 0.001). (B) The NF-κB TFBM in the promoter sequence of *SLC7A5* (-159, -211, -396, -507, -605, -692, -737, -782, -1073, -1284, -1322, -1371, -1573, p < 0.001). (C) The STAT5 TFBM in the promoter sequence of *SLC1A3* (-1432, -1254, -1005, -844, -442, -354, -114, 75, p < 0.001). (D) The STAT5 TFBM in the promoter sequence of *SLC7A5* (-1018, -1067, -1206, -1768, p < 0.001). (E) The TFBM of NF-κB of Jaspar Database. (F) The TFBM of STAT5 of Jaspar Database.

Table S1 The target genes and primers for qPCR

Name	Sequence (5′- 3′)
SLC1A3 (NM_174600.2)	P1:GGAGAGATTCCAGCAAGG<br>P2:TTCTGTATGGTCGGAGGGT
SLC7A5 (NM_174613.2)	P1:GCCGTGTGTGGCGTGTTCTC<br>P2:GTAGGTGGCGAAGACGAGCG
ACTB (NM_173979.3)	P1:CACCACGGCCGAGCGGGAAATC<br>P2:AGAGCCTCAGGGCAGCGGAACC

Table S2 The colon number of *S. aureus* in BMECs and cells medium（$\bar{x}$ ±SD）

Infection time		2 h	4 h	8 h
S. aureus （CFU/mL）	cell medium	0	0	0
	cell lysate	(1.50±0.2)×10^3	(3.03±0.25)×10^3	(5.13±0.8)×10^3
